# Indole diketopiperazines from endophytic *Chaetomium sp 88194* induce breast cancer cell apoptotic death

**DOI:** 10.1038/srep09294

**Published:** 2015-03-19

**Authors:** Fu-qian Wang, Qing-yi Tong, Hao-ran Ma, Hong-feng Xu, Song Hu, Wei Ma, Yong-bo Xue, Jun-jun Liu, Jian-ping Wang, Hong-ping Song, Jin-wen Zhang, Geng Zhang, Yong-hui Zhang

**Affiliations:** 1 Department of Pharmacy, Wuhan First Hospital, Wuhan 430022, Hubei, People's Republic of China; 2 Hubei Key Laboratory of Natural Medicinal Chemistry and Resource Evaluation, School of Pharmacy, Tongji Medical College, Huazhong University of Science and Technology, Wuhan 430030, People's Republic of China; 3 Tongji Hospital Affiliated to Tongji Medical College, Huazhong University of Science and Technology, Wuhan 430030, People's Republic of China; 4 Puai Hospital Affiliated to Tongji Medical College, Huazhong University of Science and Technology, Wuhan 430030, People's Republic of China

## Abstract

Diketopiperazines are important secondary metabolites of the fungi with variety bioactivities. Several species belonging to genus *Chaetomium* produce compounds of this class, such as chetomin. To identify new antitumor agents, secondary metabolites of fungus *Chaetomium sp 88194* were investigated and three new indole diketopiperazines, Chaetocochins G (1), Oidioperazines E (2) and Chetoseminudin E (3), along with two known compounds Chetoseminudins C (4) and N-acetyl-*β*-oxotryptamine (5), were obtained. Chaetocochins G and Chetoseminudin E were recrystallized in CHCl_3_ containing a small amount of MeOH, and their structures with absolute configuration were established by spectroscopic data interpretation and single-crystal X-ray diffraction analysis. The absolute configuration of Oidioperazines E was defined by comparing of experimental and calculated electronic circular dichroism spectra. These isolates were also evaluated the anticancer activity, and Chaetocochins G displayed more potent cytotoxicity in MCF-7 cells than the common chemotherapeutic agent (5-fluorouracil) associated with G2/M cell cycle arrest. More importantly, Chaetocochins G induced cell apoptotic death via caspase-3 induction and proteolytic cleavage of poly (ADP-ribose) polymerase, concomitantly with increased Bax and decreased Bcl-2 expression. Our findings suggested that indole diketopiperazines from endophytic *Chaetomium sp 88194* may be potential resource for developing anti-cancer reagents.

Endophytic fungi metabolites have gained increased attention as a new resource for the discovery of new therapeutic agents^1^,^2^. The endophytic fungi of the genus *Chaetomium* produce many types of secondary metabolites, such as indole diketopiperazines which are widely found as mold secondary metabolites with bioactivities including antitumor, antimicrobial, antinematodal, and cytotoxicity effects^3^.

Breast cancer is one of four oncology diseases that are most widespread in the world, it also one of leading causes of cancer-related deaths in female population^4^, causing death of about 350,000 women in both developed and developing countries every year^5^.

In order to find new antitumor agents, the extracts of the fermented rice substrate of the fungus *Chaetomium cochliode*s *88194* that obtained from China Forestry Culture Collection Center (cfcc 88194) was investigated, leading to the isolation of three new indole diketopiperazines (**1**–**3**) and two known compounds (**4**–**5**)^6^,^7^.Our work thereby provides three new indole diketopiperazines (**1**, **2**, and **3**), and Chaetocochins G (**1**) display antiproliferative effect, at least partially, due to the induction of apoptosis in MCF-7 cells.

## Results and Discussion

Our present research is focused on determining the structures of indole diketopiperazines (**1**–**5**) from *Chaetomium cochliode*s *88194* and investigating the antiproliferative effect of isolates against the MCF-7 human breast cancer cell line. The results showed that Chaetocochins G exhibited cytotoxicity with IC_50_ values of 8.3 *μ*g/mL (48 h). Cell cycle arrest is regarded as one of effective strategies for eliminating the cancer cells^8^,^9^. Besides cell cycle, apoptosis, acting as a protective mechanism that destroys potentially harmful or damaged cells before manifestation of malignancy^10^, is also another key strategy for eliminating cancerous cells^11^,^12^. Chaetocochins G significantly inhibits proliferation in MCF-7 cell lines, with the observation of cell cycle arrest at the G2/M phase and activated cellular apoptosis through activation of caspase-3 and proteolytic cleavage of poly (ADP-ribose) polymerase. It also could increase Bax and decrease Bcl-2 expression.

### Structure elucidation

Compound **1** was isolated as colorless needle crystals. Its molecular formula, C_35_H_42_N_6_O_6_S_4_, was established by HRESIMS (*m/z* 793.1934 [M + Na]^+^, calcd. 793.1941). Its IR spectrum showed characteristic absorptions of hydroxyl group (3399 cm^−1^), and four carbonyl groups (1605, 1632, 1647 and 1667 cm^−1^).

**Figure 1:**
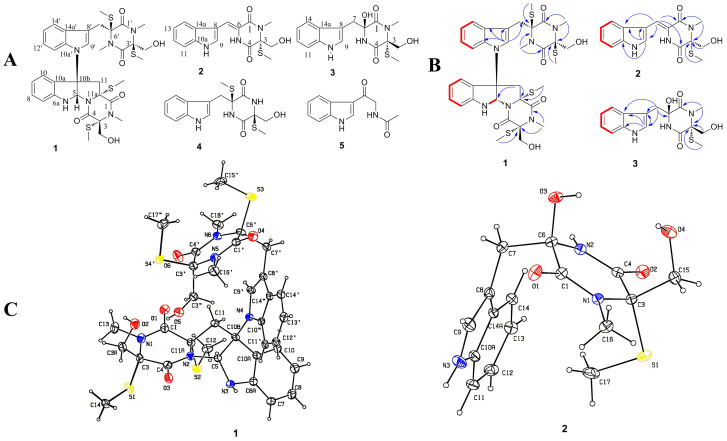
Chemical structures of the isolated compounds. (A) Isolated compounds from extracts of fungus *Chaetomium sp 88194* fermented rice substrate. (**1**–**5**). (B) Selected ^1^H−^1^H COSY (red bold lines), and HMBC (H**→**C) correlations. (C) ORTEP drawing of Chaetocochins G (**1**) and **3**.

The ^1^H NMR spectrum of **1** (Table 1) exhibited signals for four S-methyl (*δ*_H_ 2.30, s; 2.24, s; 2.10, s; 2.00, s), three N-methyl (*δ*_H_ 3.20, s; 3.12, s; 2.78, s), four methylene groups (*δ*_H_ 3.18/3.88, d; 3.34/3.58, d; 3.84/4.32, d; 3.09/3.74, d), two sets of aromatic protons (*δ*_H_ 6.73/7.04, d; 7.25/6.94, t; 6.87/7.03, t; 7.27/7.57, d), and an olefinic protons (*δ*_H_ 7.01, s). Correspondingly, the ^13^C NMR and DEPT spectra showed the presence of the S-methyl, N-methyl, aromatic and olefinic carbons, together with five sp^2^ quaternary carbons (*δ*_C_ 149.8, 127.9, 134.8, 129.6, 107.6), five sp^3^ quaternary carbons (*δ*_C_ 68.6, 71.3, 72.9, 73.2, 73.4), one methines bearing heteroatoms (*δ*_C_ 81.7), and four carbonyl groups (*δ*_C_ 164.2, 165.9, 166.0, 166.6). In light of the above data, compound **1** was determined to be a chetomin-type indole diketopiperazine^13^,^14^ and should be an analogue of dethio-tetra (methylthio) chetomin^15^. The planar structure of **1** was deduced unambiguously from analysis of the 2D-NMR spectroscopic data, including ^1^H−^1^H COSY, HSQC, and HMBC experiments (Fig. 1A and 1B). However, no NOESY correlations were observed that enabled relative configuration of **1**.

Although it is hard to determine configuration of tryptophan–derived epidithiodioxopiperazines for crystallization is difficult, **1** was recrystallized in CHCl_3_ containing a small amount of MeOH, so the crystal of compound **1** was subjected to single-crystal X-ray diffraction analysis with Cu K*α* radiation, which unambiguously confirmed the structure. The result indicated a 3*R*, 5*S*, 10b*R*, 11a*R*, 3**′***S* and 6**′***S* absolute configuration (Fig. 1C) and **1** was named as Chaetocochins G.

Chaetocochins G (**1**), colorless needle crystals; mp 209.6°C; 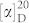 + 198 (*c* = 0.61, CDCl_3_); UV (CHCl_3_) *λ*_max_ (log *ε*) 217 (4.79), 295 (4.06) nm; IR (KBr) *ν*_max_ 3399, 3327, 2919, 1667, 1647, 1632, 1605, 1421, 1385, 1058, 751 cm^−1^; CD (CDCl_2_) 303 ([*θ*], +54169), 257 ([*θ*], +29483) nm; ^1^H and ^13^C NMR data, see Table 1; (+)-HRESIMS *m/z* 793.1934 [M + Na]^+^ (calcd for C_35_H_42_N_6_NaO_6_S_4_^+^, 793.1941).

Compound **2** was isolated as a yellow amorphous powder. The sodiated molecular ion peak at *m/z* 354.0883 [M + Na]^+^ (calcd 354.0883) observed in the HRESIMS corresponded to a molecular formula of C_16_H_17_N_3_O_3_S. The IR absorption bands at 1639, 1674 and 3429 cm^−1^ implied the presence of hydroxyl group and carbonyl groups. Comparison of ^1^H NMR data (Table 2) indicated that **2** had the same scaffold as oidioperazine C^16^. The absence of the methoxyl signal at *δ*_H_ 3.15, together with the presence of an additional S-methyl (*δ*_H_ 2.08) and a N-methyl (*δ*_H_ 3.24), indicated the structure of **2** as shown (Fig. 1A). The cross-peaks of H-NCH_3_ (*δ*_H_ 3.24) to C-1 (*δ*_C_ 161.7) and C-3 (*δ*_C_ 75.0) in the HMBC spectrum of **2** indicated that the N-methyl group was located at N-2. Similarly, the S-methyl group (*δ*_H_ 2.08) was located at C-3 (*δ*_C_ 75.0) (Fig. 1B).

**Figure 2:**
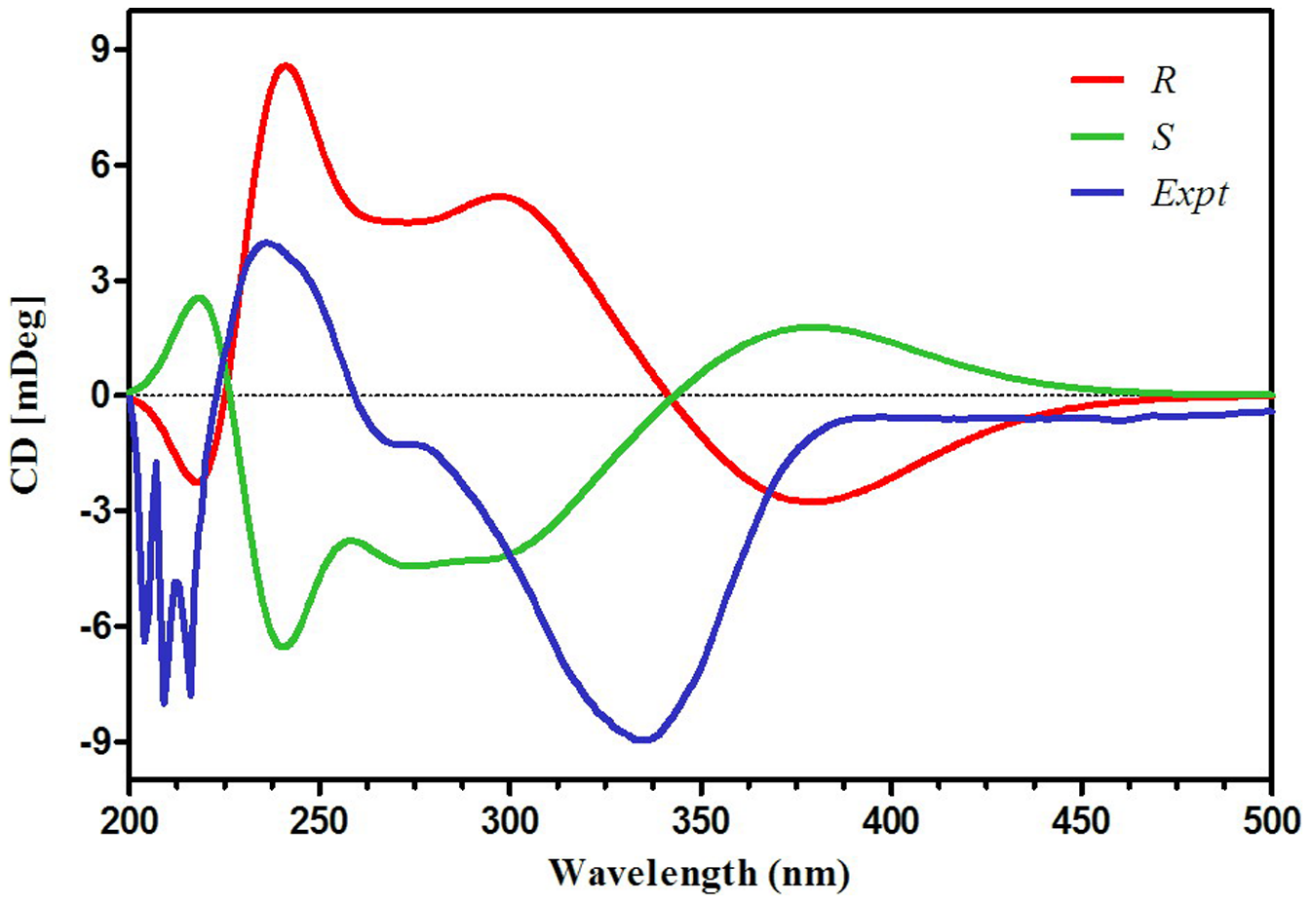
Comparison of experimental and calculated ECD spectra of Oidioperazines E (2) and the stereoisomers (3*S*) and (3*R*).

The absolute configuration of **2** was determined by comparing its experimental and calculated ECD spectra. The cotton effects in the experimental ECD spectrum were consistent with those in calculated ECD curve of (3*R*)-2 (Fig. 2). So, the structure of **2** was determined to be 3*R* configuration and named as Oidioperazines E.

Oidioperazines E (**2**): yellow amorphous powder; 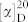 − 190 (*c* = 2.07, CDCl_3_); UV (CHCl_3_) *λ*_max_ (log *ε*) 227 (4.24), 339 (4.16) nm; IR (KBr) ν_max_ 3429, 2928, 1674, 1639, 1530, 1432, 1103, 740 cm^−1^; CD (CDCl_2_) 236 ([*θ*], +35236), 335 ([*θ*], −79145) nm; ^1^H NMR and ^13^C NMR data, see Table 2; (+)-HRESIMS *m/z* 354.0883 [M + Na]^+^ (calcd for C_16_H_17_N_3_NaO_3_S^+^, 354.0883).

Compound **3** was isolated as colorless cubic crystals. The molecular formula of **3** was determined to be C_16_H_19_N_3_O_4_S by HRFABMS (*m/z* 372.0988 [M + Na]^+^, calcd. 372.0986). The IR spectrum of **3** gave the absorption of hydroxyl group (3325 cm^−1^), and carbonyl groups (1685 and 1645 cm^−1^).

The ^1^H NMR data of **3** (Table 2) exhibited resonances for monosubstituted indole system at *δ*_H_ 7.70 d, 7.00 dd, 7.05 dd, 7.27, and 7.08 s, a S-methyl at *δ*_H_ 0.64, a N-methyl at *δ*_H_ 2.94, two methylene groups at *δ*_H_ 3.12/3.75 and 3.56/3.82. Analogous resonances consistent with the presence of these functionalities appeared in the ^13^C NMR data of **3** (Table 2). According to the DEPT and HSQC spectra analysis of **3**, two sp^3^ quaternary carbons bearing heteroatoms (*δ*_C_ 77.7, 84.1) and two carbonyl groups (*δ*_C_ 167.9, 170.3) could be observed.

The S-methyl moiety was located at the C-3 positions, as supported by the HMBC correlations (Fig. 1B) between H-SCH_3_/C-3. N-methyl assigned at the C-2 positions from the HMBC correlations between H-NCH_3_ from C-3 and C-1. Therefore, the planar structure of **3** is determined to be an analogue of Chetoseminudins B^4^.

As mentioned above, no NOESY correlations were observed to determine the relative configuration of **3**. The absolute configuration of **3** was determined by the X-ray single crystal diffraction analysis with Cu K*α* radiation. The results indicated that the crystal structure of compound **3** is shown in Figure 1C, and its absolute configuration was determined as 3*R*, 6*S* and named Chetoseminudin E.

Chetoseminudin E (**3**): colorless cubic crystals; mp 193.3°C; 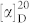 +54 (*c* = 0.64, CH_3_OH); UV (CHCl_3_) *λ*_max_ (log *ε*) 219 (4.66), 281 (3.89) nm; IR (KBr) ν_max_ 3349, 3325, 2920, 1685, 1645, 1427, 1397, 1242, 1056, 752 cm^−1^; CD (CDCl_2_) 267 ([*θ*], +24809), 299 ([*θ*], −4996) nm; ^1^H NMR and ^13^C NMR data, see Table 2; (+)-HRESIMS *m/z* 372.0988 [M + Na]^+^ (calcd for C_16_H_19_N_3_NaO_3_S_2_^+^, 372.0986).

### Chaetocochins G (1) causes proliferation inhibition and G2/M phase arrest in MCF-7 cells

**Figure 3:**
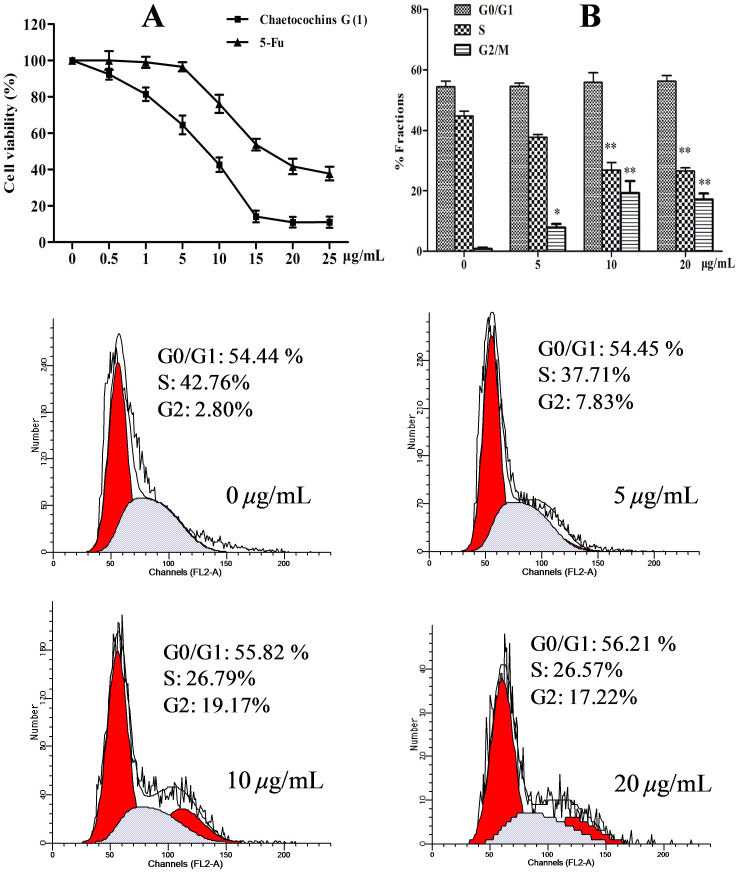
Chaetocochins G (1) causes inhibitory proliferation and G2/M phase arrest in MCF-7 cells. (A) Chaetocochins G inhibits the proliferation of MCF-7 cells. Cells were treated with various concentrations of Chaetocochins G for 48 h. Cell viability was detected using MTT assay, 5-Fu was used as positive control. (B) Cell cycle progression of Chaetocochins G in MCF-7 Cells. Data are presented as the means ± SD of three experiments, *P < 0.05, **P < 0.01 compared to the control group.

All compounds were evaluated for cytotoxic effect against MCF-7 cells, together with one noncancerous human pulmonary epithelial cell line BEAS-2B, using MTT method^17^. 5-fluorouracil (5-Fu) was selected as positive control. As showed in Figure 3A, Chaetocochins G exhibited more potent cytotoxicity than 5-Fu (IC_50_ values was 16.9 *μ*g/mL). The IC_50_ values of 48 h against MCF-7and Beas-2B cell were 8.3, and 9.7 *μ*g/mL. Compounds **2**, **3**, **4** and **5** did not show obvious cytotoxicity (IC_50_ >40 *μ*g/mL). So Chaetocochins G (**1**) was chosen for a further investigation.

Cell cycle is important for the proliferation of cancer^18^. To investigate cell cycle perturbations in MCF-7 cells induced by Chaetocochins G, flow cytometric analysis was performed after treatment at various concentrations (0, 5, 10, and 20 *μ*g/mL) for 48 h. The results (Fig. 3B) revealed that Chaetocochins G arrested the cell cycle at the G2/M phase in MCF-7 cells in a concentration-dependent manner, which induced a corresponding increase percentage of cells in the G2/M-phase (7.83%, 19.17%, and 17.22%, compared to 2.8% in untreated cells) and decrease in the S-phase (37.71%, 26.79%, and 26.57%, compared to 42.76% in untreated cells).

### Morphological changes of MCF-7 cells treated with Chaetocochins G

**Figure 4:**
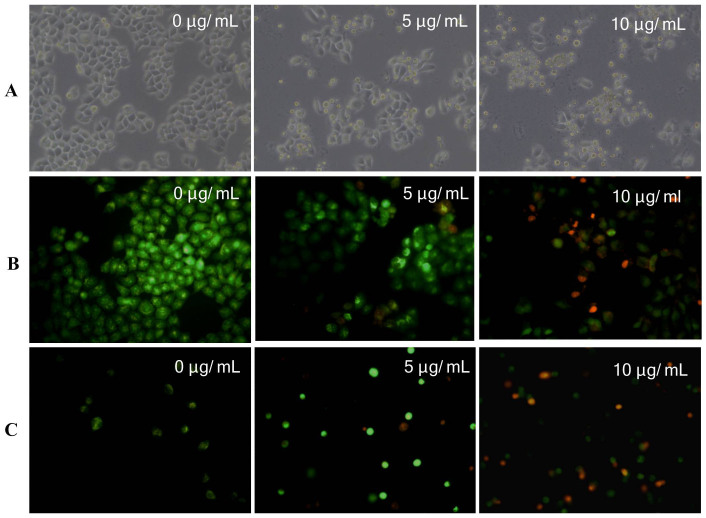
Morphological changes of MCF-7 cells induced by Chaetocochins G. (A) Cells shrinkage and abnormal morphological features could be observed (100×). (B) Cells on coverslips treated with Chaetocochins G at 5.0 and 10 *μ*g/mL, stained with AO/EB (100×). (C) Cells in suspension treated with Chaetocochins G at 5.0 and 10 *μ*g/mL, stained with AO/EB (100×).

The morphological changes were examined using an invert light microscope as well as a fluorescence microscope after acridine orange (AO) and ethidium bromide (EB) double staining^19^. As shown in Figure 4A, cell shrinkage and abnormal morphological features could be observed in 5.0, and 10.0 *μ*g/mL Chaetocochins G treated groups. What's more, shrunk or colored orange cells that indicated apoptosis^20^ were observed both on the coverslips and suspension during fluorescence microscope examination (Figure 4B, 4C). On the contrary, the untreated cells exhibited large green nuclei indicating the intact cell membranes.

### Chaetocochins G (1) causes MCF-7 cell death by apoptosis

To determine whether Chaetocochins G meditated inhibition of growth and proliferation was associated with apoptosis, the flow cytometry apoptosis assay was performed using Annexin V and PI staining^20^,^21^,^22^,^23^.

**Figure 5:**
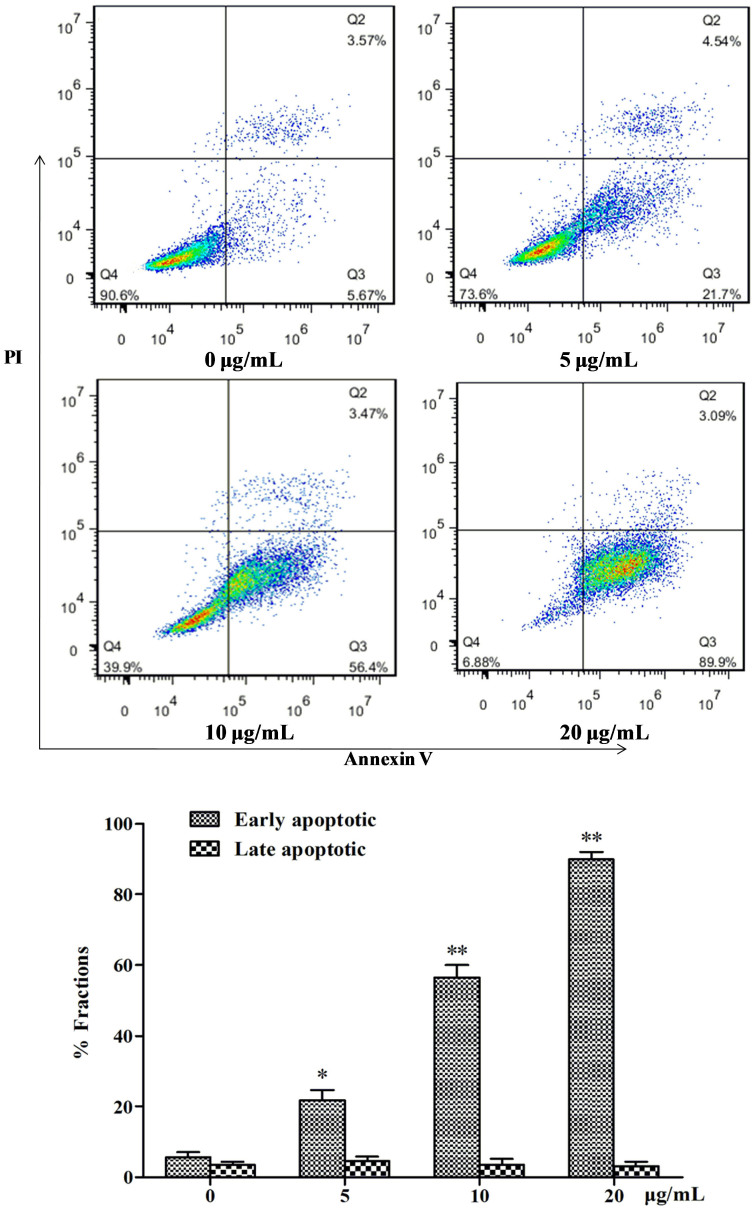
Analysis of apoptosis in Chaetocochins G treated MCF-7 Cells. Cells were treated with Chaetocochins G at various concentrations (0, 5, 10, and 20 *μ*g/mL) for 48 h, then collected and stained with annexin V/PI, and subjected to flow cytometry. Data are presented as the means ± SD of three experiments. *P < 0.05, **P < 0.01 compared to the control group.

After treatment at various concentrations (0, 5, 10, and 20 *μ*g/mL) for 48 h, the apoptosis of the cells were detected. As shown in Figure 5 the apoptotic cells accumulated in a dose-dependent manner from 26.24% to 92.99%. These results demonstrate that Chaetocochins G induced apoptosis of MCF-7 cells.

### Chaetocochins G (1) changes the expression of apoptotic-related proteins

Among the caspase-family, caspase-3 is considered to be a critical effector and the activation of caspase-3 could result in PARP degradation^24^. Bak and Bax have been categorized as prodeath members of Bcl-2 family. Protein, such as cytochrome c, could release into cytoplasm from the mitochondria when the Bak and Bax homo-oligomerization on the mitochondrial membrane. Meanwhile, antiapoptotic Bcl-2 members could prevent mitochondrial protein release by interacting with and inhibiting both Bak and Bax^25^.

**Figure 6:**
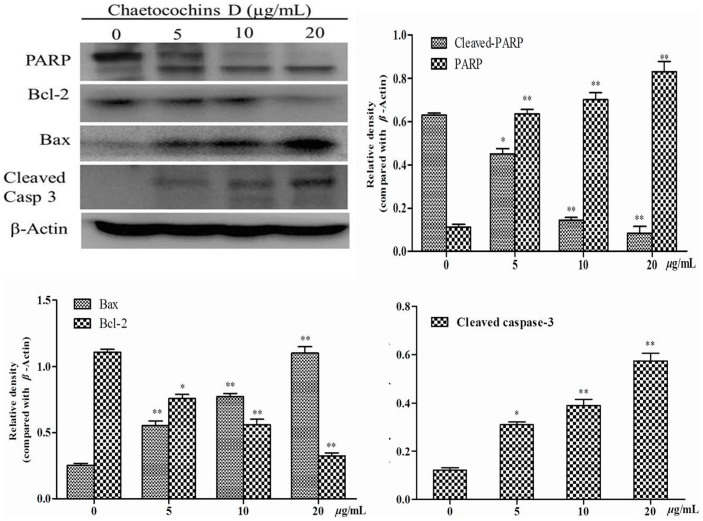
Changes of apoptosis-related protein in MCF-7 cells after Chaetocochins G treated. The relative density was detected by Image J, data are presented as the means ± SD of three experiments, significant differences from the control value (0 *μ*g/mL) are indicated by *p < 0.05, **p < 0.01.

MCF-7 cells were treated with Chaetocochins G as describe above. Changes of apoptosis-related protein, including Bax, Bcl-2, cleaved caspase-3 and PARP, were assessed by western blot analysis. *β*-actin was used as an internal control and relative optical density of proteins were compared with *β*-actin. As shown in Figure 6, the Bax protein levels increased dose dependently. In contrast, anti-apoptotic protein Bcl-2 declined upon Chaetocochins G treatment. Chaetocochins G**-**induced activation of caspase-3 was evidenced by appearance of cleaved caspase-3 and PARP (poly ADP-ribose polymerase)^26^ in a concentration-dependent manner. All of the results above indicated that Chaetocochins G could induce apoptosis in MCF-7 cells.

In conclusion, the current study describes the isolation and identification of three new indole diketopiperazines (**1–3**) and two known compounds (**4**, **5**) from the extracts of *Chaetomium sp 88194*. All the compounds were evaluated for the cytotoxicity against MCF-7 cell line. The results indicated that Chaetocochins G (**1**) displayed much more potent inhibitory activity than the common chemotherapeutic agent 5-fluorouracil, with IC_50_ values of 8.3 *μ*g/mL, and induced cell cycle arrest at the G2/M phase in MCF-7 cells, accompanied by initiation of apoptosis. Further study demonstrated that Chaetocochins G induced cellular apoptosis via the activation of the Caspase 3, PARP, and Bax up-regulation as well as Bcl-2 inhibition. This paper is the first report for apoptosis-inducing effects of tryptophan–derived epidithiodioxopiperazines, and provides an insight into their anticancer mechanism. These results suggest that indole diketopiperazines from endophytic *Chaetomium sp 88194* may be potential resource for anticancer drug research and discovery.

## Methods

### General experimental procedures

Melting points (uncorrected) were determined on a Beijing Tech X-5 microscopic melting point apparatus (Beijing Tech Instrument Col. Ltd, Beijing, China). Optical rotations were determined in CHCl_3_ or MeOH on a Perkin-Elmer 341 polarimeter. UV and FT-IR spectra were determined using PerkinElmer Lambda 35 and Bruker Vertex 70 instrument, respectively. HRESIMS was performed on a Thermo Scientific LTQ-XL mass spectrometer. NMR spectra were recorded on a Bruker AM−400 NMR spectrometer and chemical shifts were referenced to the solvent peaks for CD_3_OD (*δ*_H_ 3.31/*δ*_C_ 49.0) or CDCl_3_ (*δ*_H_ 7.26/*δ*_C_ 77.16). The CD spectra were obtained on a JASCO J-810 spectrometer. X-ray crystallographic data were collected from Bruker Smart APEX-II CCD diffractometer equipped with graphite-monochromatized Cu K*α* radiation (*λ* = 1.54178 Å). HPLC isolation was performed on waters 2535 pump and a waters 2998 detector using a C_18_ column (5 *μ*m, 10 × 250 mm, SunFire™ Prep C_18_) and MeOH−H_2_O or CH_3_CN−H_2_O as the mobile phase at 2.0 mL/min flow rate. MPLC was carried out with a QuikSep-50 chromatography system (H&E Co., Ltd, Beijing, China). TLC was carried out using glass-precoated silica gel GF254 (Qingdao Marine Chemical, Inc., Qingdao, China) and visualized under UV light. Silica gel (100–200 mesh and 200–300 mesh, Qingdao Marine Chemical Inc., Qingdao, China), ODS (50 *μ*m, YMC, Kyoto, Japan), and Sephadex LH−20 (Pharmacia Biotech AB, Uppsala, Sweden) were used for column chromatography.

### Fungal strain and identification

The strain of the fungus obtained from China Forestry Culture Collection Center (CFCC, Beijing, China) was isolated from the *Cymbidium goeringii*, which was collected from Xinning, Hunan Province of China, in November 2008. The strain was purified and identified belonging to the *Chaetomium sp* by molecular identification. The DNA sequence data from the fungus were deposited at GenBank with accession number KP171518, and the strain was deposited at Hubei Key Laboratory of Natural Medicinal Chemistry and Resource Evaluation, School of Pharmacy, Tongji Medical College, Huazhong University of Science and Technology, Wuhan, PR China.

### Fermentation and isolation

The fermentation was carried out in 40 fernbach flasks (1000 mL), each containing 200 g of rice and distilled H_2_O (200 mL). After autoclaving at 121°C, 15 psi for 30 min, each flask cooling to room temperature was inoculated with the fresh mycelium and incubated at 25°C for 28 days.

The fermented solid rice medium (8.0 kg) was soaked with 95% aqueous EtOH (40 L × 3, 1 day for each time) at room temperature. The solvent was evaporated in vacuo to afford a residue (22.0 g), which was then partitioned between ethyl acetate (3 L × 3) and aqueous (3 L). On evaporation, the organic phase (250 g) was subjected to silica gel (200–300 mesh) column chromatography and eluted with a MeOH−CHCl_2_ gradient to give four fractions, A–D. Fractions C (2.7 g) was chromatographed over ODS by MPLC and eluted with a MeOH−H_2_O gradient to yield four fractions (C_A_–C_D_).

Subfraction C_A_ was further separated by semipreparative HPLC (CH_3_CN–H_2_O, 60:40, flow rate: 2 mL/min) to yield compounds **1** (67.31 mg, t*_R_* 20.3 min). Subfraction C_B_ was further separated over Sephadex LH-20 eluting with CHCl_3_–MeOH (1:1) to give three subfractions (C_BA_–C_BC_). Subfraction C_BB_ was further purified by semi-preparative HPLC eluted with MeOH–H_2_O (40:60, flow rate: 2 mL/min) to give compounds **3** (8.5 mg, t*_R_* 28.9 min), compounds **2** (10.37 mg, t*_R_* 28 min) was obtained from C_BC_ through semi-preparative HPLC eluted with CH_3_CN–H_2_O (65:35, flow rate: 2 mL/min). Subfraction C_C_ was purified by Sephadex LH-20 column chromatography eluting with CHCl_3_–MeOH (1:1) to give two subfractions (C_CA_–C_CB_). Then, C_CB_ was chromatographed over ODS by MPLC and eluted with a MeOH−H_2_O gradient to yield compounds 4 (1.95 mg) and 5 (5.8 mg).

### Crystallographic Data and X-ray Structure Analysis

Colorless needle crystals of Chaetocochins G (**1**) were obtained from CHCl_3_ containing a small amount of MeOH at room temperature. Crystal data were obtained on a Bruker Smart APEX-II CCD single-crystal X-ray diffractometer with the Cu K*α* (*λ* = 1.54178 Å). Structure solution and refinement were performed with the SHELXL-97. Crystal data of **1**: C_35_H_42_N_6_O_6_S_4_·H_2_O (M = 789.00); needle crystal (0.20 × 0.20 × 0.10 mm); space group *P*21; unit cell dimensions *a* = 13.3191(3) Å, *b* = 13.7927(3) Å, *c* = 21.8874(5) Å, *α* = 90.00°, *β* = 96.0450(10)°, *γ* = 90.00°, *V* = 3998.50(15) Å^3^; Z = 4; *T* = 296(2) K; *ρ*_calcd_ = 1.311 Mg/m^3^; absorption coefficient 2.623 mm^−1^, *F*(000) = 1664, A total of 10529 reflections were collected in the range 2.03° < θ < 58.42° with 10529 independent reflections [*R*(int) = 0.0000]; completeness to *θ*_max_ was 97.3%; full-matrix least-squares refinement on *F*^2^; the number of data/parameters/restraints were 10529/1029/47; goodness-offit on *F*^2^ = 1.067; final *R* indices [*I* > 2*σ*(*I*)] *R*_1_ = 0.0437, *wR*_2_ = 0.1226; *R* indices (all data) *R*_1_ = 0.0578, *wR*_2_ = 0.1312; absolute structure parameter 0.015(12).

Colorless cubic crystals of Chetoseminudin E (**3**) were obtained from CH_3_OH at room temperature. Crystal data were obtained on a Bruker Smart APEX-II CCD single-crystal X-ray diffractometer with the Cu K*α* (*λ* = 1.54178 Å). Structure solution and refinement were performed with the SHELXL-97. Crystal data of **3**: 2(C_16_ H_20_ N_3_ O_4_ S)·H_2_ O (M = 716.82); monoclinic crystal (0.71 × 0.53 × 0.40 mm); space group *P*21; unit cell dimensions *a* = 8.8404(2) Å, *b* = 14.4203(3) Å, *c* = 13.5625(2) Å, *α* = 90.00°, *β* = 92.8240(10)°, *γ* = 90.00°, *V* = 1726.86(6) Å^3^; Z = 2; *T* = 298(2) K; *ρ*_calcd_ = 1.379 Mg/m^3^; absorption coefficient 1.925 mm^−1^, *F*(000) = 756, A total of 21876 reflections were collected in the range 3.26° < θ < 68.72° with 6000 independent reflections [*R*(int) = 0.0328]; completeness to *θ*_max_ was 96.9%; full-matrix least-squares refinement on *F*^2^; the number of data/parameters/restraints were 6000/452/1; goodness-offit on *F*^2^ = 1.042; final *R* indices [*I* > 2*σ*(*I*)] *R*_1_ = 0.0284, *wR*_2_ = 0.0785; *R* indices (all data) *R*_1_ = 0.0286, *wR*_2_ = 0.0787; absolute structure parameter 0.025(10).

Crystallographic data for the structures of Chaetocochins G (**1**) and Chetoseminudin E (**3**) have been deposited in the Cambridge Crystallographic Data Centre [deposition numbers: CCDC 1029222 (**1**), and CCDC 1029221 (**3**)]. Copies of these data can be obtained free of charge at www.ccdc.cam.ac.uk/conts/retrieving.html (or from the Cambridge Crystallographic Data Centre, 12 Union Road, Cambridge CB21EZ, UK; fax: (+44) 1223-336-033; or e-mail: deposit@ccdc.cam.ac.uk).

### Computational methods for ECD spectra

The conformational spaces for the 3*R* and 3*S* isomers of Oidioperazines E (**2**) were explored using both BALLOON^27^ and confab^28^ programs. The BALLOON program searches conformational space with genetic algorithm, whereas the confab program systematically generates diverse low energy conformations that are supposed to be close to crystal structures. Duplicate conformations were identified and removed when the root-mean-square (RMS) distance was less than 0.5 Å. The semi-empirical PM3 quantum mechanical geometry optimizations were performed on the conformations by using Gaussian 09 program. The remaining conformations were further optimized at B3LYP/6-31G* level of theory in dichloromethane solvent with IEFPCM3^29^ solvation model by using Gaussian 09 program, and the duplicated conformations emerging after this calculations were removed according to the same RMS criteria above. The harmonic vibrational frequencies were performed to confirm the stability of the finally obtained conformers. The oscillator strengths and rotational strengths of 20 weakest electronic excitations of each conformer were calculated using the TDDFT methodology at the B3LYP/6-311++G** level of theory with dichloromethane as solvent by the IEFPCM solvation model implemented in Gaussian 09 program. The ECD spectra for each conformer were then simulated by using a Gaussian function with a bandwidth *σ* of 0.45 eV. The calculated spectra for each conformation were combined after Boltzmann weighting according to their population contribution.

### Cell culture and cell viability assay

MCF-7 human breast cancer cells, purchased from the cell bank of the Basic Medical College of Huazhong University of Science and Technology, were cultured in DMEM medium (Solarbio, Peking, People's Republic of China), supplemented with 10% fetal bovine serum (Sijiqing, Hangzhou, People's Republic of China), 100 U/mL penicillin, and 100 *μ*g/mL streptomycin. Cultures were maintained in an incubator (Thermo Fisher Scientific, Marietta, USA) with 5% CO_2_ at 37°C.

The viability of cells was performed using an MTT [3-(4,5-dimethylthiazol-2-yl)-2,5-diphenyltetrazolium bromide] (Biosharp, Hefei, People's Republic of China) method in 96-well microplates, as reported previously^30^, with slight modification. Cells were plated in 96-well culture plates (9 × 10^3^ per well) and allowed to adhere for 12 h before treated with Chaetocochins G at a series of concentration (0, 0.5, 1, 5, 10, and 20 *μ*g/mL) for 48 hours. Add 20 *μ*L MTT (5 mg/mL) per well was added prior to 4 h incubation at 37°C. Upon medium removal, formazans depositing on the plate were dissolved in 150 *μ*L DMSO, then, the cell viability was detected by a Thermo Multiskan MK3 microplate reader (Thermo Scientific, Helsinki, Finland) at *λ* = 492 nm, and the IC_50_ values were obtained from the MTT viability curves using GraphPad Prism 4.0.

### Cell morphology

After incubation with various concentrations of Chaetocochins G (0, 5, and 10 *μ*g/mL) for 48 h. a staining method using AO (Solarbio, Peking, People's Republic of China) and EB (Solarbio, Peking, People's Republic of China) was performed and photographed using a fluorescence microscope (Olympus BX-51, Tokyo, Japan).

### Cell cycle analysis

The cell cycle analysis was detected with a cell cycle and apoptosis analysis kit (Beyotime, Haimen, People's Republic of China). MCF-7 cells were exposed to Chaetocochins G (0, 5, 10 and 20 *μ*g/mL) and incubated for 48 h. Then, the cells were harvested and washed with ice-cold PBS buffer, fixed with 70% alcohol at 4°C for 12 h, and stained with propidium iodide (PI) in the presence of 1% RNAase A. After 15 min incubation at 37°C, the cells were analyzed by a flow cytometry (Becton Dickinson, San Jose, USA). The gated data were analyzed by modfit softwares (Verity Software House, Topsham, Maine, USA).

### Flow cytometric and western blotting analysis of cell apoptosis

Apoptosis assay was performed using an apoptosis detection kit (Keygen, Nanjing, People's Republic of China) according to the manufacturer's instructions. Briefly, after exposure to Chaetocochins G (0, 5, 10 and 20 *μ*g/mL) for 48 h, cells were collected, washed with annexin-binding buffer, incubated with annexinV-FITC/PI for 15 min and analyzed by a flow cytometry (Becton Dickinson, San Jose, CA, USA).

### Western blotting

Western blotting analysis was performed as described^31^. MCF-7 cells were treated with Chaetocochins G in 0, 5, 10 and 20 *μ*g/mL. After 48 h, cells were harvested and washed with ice-cold PBS and then lysed in ice cold RIPA lysis buffer for 30 min. The lysate was centrifuged at 12000 rpm for 15 min at 4°C and the supernatant was collected, the total protein concentration was determined using the BCA assay; the proteins were then dissolved in SDS sample buffer and denatured. Proteins were separated using 10% SDS–PAGE, and then transferred to PVDF (polyvinylidene fluoride) (Bio-Rad, Hercules, CA) membranes. After incubated overnight at 4°C with the respective primary antibodies, the horseradish peroxidase-conjugated secondary antibodies were added. Finally, the reactive band was identified using an enhanced chemiluminescent substrate to horseradish peroxidase.

### Statistical analysis

Statistical analysis of the data was processed with GraphPad Prism 4.0 software. Statistical analysis of the data was expressed as mean ± SD. Values were analyzed by one-way analysis of variance (ANOVA) using SPSS version 12.0 software. *p* < 0.05 were considered statistically significant.

## Author Contributions

Y.H.Z., G.Z. and Y.B.X. designed experiments. J.J.L. complete the computation section. W.M. and J.W.Z. wrote the paper. Q.Y.T. and H.R.M. conducted experiments. F.Q.W. isolated and identified of the compounds. H.P.S. analyzed data. J.P.W., H.F.X. and S.H. prepared the article in the journal's format. All authors reviewed the manuscript.

## Supplementary Material

Supplementary Information Supporting information

Supplementary Information CIF check of Chaetocochins G (1)

Supplementary Information CIF check of Chetoseminudin E (3)

## Figures and Tables

**Table 1:** NMR data [CDCl_3_, 400 MHz (^1^H), 100 MHz (^13^C)] for Chaetocochins G

position	*δ*_C_	*δ*_H_ (*J* in Hz)
1	166.04 s	
2-NCH_3_	30.86 q	3.20 s
3	73.43 s	
3-SCH_3_	14.49 q	2.30 s
3-CH_2_OH	64.41 t	3.84 m
		4.32 d (8.06)
4	164.24 s	
5	81.7 d	6.14 d (2.40)
6		5.22
6a	149.8 s	
7	110.4 d	6.73 d (6.87)
8	131.06 d	7.25 t (7.61)
9	119.8 d	6.87 td (7.49, 0.6)
10	124.61 d	7.27 d (8.12)
10a	127.9 s	
10b	72.97 s	
11	44.7 t	3.34 d (14.30)
		3.58 d (14.30)
11a	68.56 s	
11a-SCH_3_	15.89 q	2.00 s
1**′**	165.96 s	
2**′**-NCH_3_	29.43 q	2.78 s
3**′**	71.3 s	
3**′**-SCH_3_	13.58 q	2.10 s
3**′**-CH_2_OH	63.98 t	3.09 d (11.85)
		3.74 dd (11.85, 6.58)
4**′**	166.62 s	
5**′**-NCH_3_	28.81 q	3.12 s
6**′**	73.15 s	
6**′**-SCH_3_	13.46 q	2.24 s
7**′**	32.8 t	3.18 d (15.17)
		3.88 d (15.17)
8**′**	107.6 s	
9**′**	125.5 d	7.01 s
10a**′**	134.8 s	
11**′**	111.9 d	7.04 d (7.69)
12**′**	122.74 d	6.94 t (7.92)
13**′**	120.15 d	7.03 t (7.57)
14**′**	119.79 d	7.57 d (7.73)
14a**′**	129.6 s	

**Table 2:** NMR data [CDCl_3_, 400 MHz (^1^H), 100 MHz (^13^C)] for Oidioperazines E (2) and Chetoseminudin E (3)

	2 in CD_3_OD		3 in CD_3_OD	
position	*δ*_C_	*δ*_H_ (*J* in Hz)	*δ*_C_	*δ*_H_ (*J* in Hz)
1	161.7 s		170.3 s	
3	75.0 s		77.7 s	
4	163.75 s		167.9 s	
6	120.66 s		84.1 s	
7	110.38 d	7.32 d (0.73)	36.9 t	3.13 d (14.0)
				3.75 d (14.0)
8	108.37 s		109.3 s	
9	125.3 d	7.77 d (0.50)	126.4 d	7.08 s
10a	136.3 s		137.9 s	
11	111.38 d	7.43 d (8.04)	120.3 d	7.70 d (7.7)
12	122.38 d	7.21 dd (8.29, 6.93)	120.1 d	7.00 dd (8.1, 7.2)
13	120.09 d	7.14 dd (8.29, 7.04)	122.5 d	7.05 dd (8.1, 7.3)
14	118.01 d	7.68d (7.84)	112.3 d	7.27 d (7.9)
14a	127.08 s		129.6 s	
2-NCH_3_	27.55 q	3.24 s	29.7 q	2.94 s
3-CH_2_OH	62.57 t	3.87 d (11.67)	64.6 t	3.82 d (11.4)
		4.27 d (11.67)		3.56 d (11.4)
3-SCH_3_	10.52 q	2.08 s	9.7 q	0.64 s
