# Supplementary material for: Indole diketopiperazines from endophytic Chaetomium sp 88194 induce breast cancer cell apoptotic death
Source: Sci Rep. 2015 Mar 19;5:9294. doi: 10.1038/srep09294 (PMC4365412; doi:10.1038/srep09294)
Supplement: Supplementary Information — Supporting information [file srep09294-s1.pdf]

Supporting Information

Indole diketopiperazines from endophytic *Chaetomium sp 88194* induce breast cancer cell apoptotic death

Fu-qian Wang[1], Qing-yi Tong[2], Hao-ran Ma[1], Hong-feng Xu[1], Song Hu[1], Wei Ma[1], Yong-bo Xue[2], Jun-jun Liu[2], Jian-ping Wang[2], Hong-ping Song[4], Jin-wen Zhang[3]*, Geng Zhang[1]*, Yong-hui Zhang[2]*

[1]*Department of Pharmacy, Wuhan First Hospital, Wuhan 430022, Hubei, People's Republic of China*
[2]*Hubei Key Laboratory of Natural Medicinal Chemistry and Resource Evaluation, School of Pharmacy, Tongji Medical College, Huazhong University of Science and Technology, Wuhan 430030, People's Republic of China*
[3]*Tongji Hospital Affiliated to Tongji Medical College, Huazhong University of Science and Technology, Wuhan 430030, People's Republic of China*
[4]*Puai Hospital Affiliated to Tongji Medical College, Huazhong University of Science and Technology, Wuhan 430030, People's Republic of China*

*Author to whom correspondence should be addressed:
Tel. +86-027–83692311(Y.H. Zhang); +86-027-8533-2089 (G. Zhang); +86-27-8366-3253 (J.W. Zhang);
Fax: +86-027-8369-1325(Y.H. Zhang); +86-027-8583-2289(G. Zhang); +86-027-8369-2762(J.W. Zhang)
Email: zhangyh@mails.tjmu.edu.cn (Y.H. Zhang); zhanggen888@126.com (G. Zhang); tjzhangjinwen@163.com (J. W. Zhang)

List of Supporting Information

Figure S1 IR spectrum of **1**
Figure S2 UV spectrum of **1**
Figure S3 (+)-HR-ESI-MS spectrum of **1**
Figure S4 ^{1}H NMR spectrum (400M) of **1** in $CDCl_3$
Figure S5 ^{13}C NMR spectrum (100M) of **1** in $CDCl_3$
Figure S6 DEPT 135 spectrum (100M) of **1** in $CDCl_3$
Figure S7 HSQC spectrum of **1** in $CDCl_3$
Figure S8 HMBC spectrum of **1** in $CDCl_3$
Figure S9 ^{1}H-^{1}H COSY spectrum of **1** in $CDCl_3$
Figure S10 NOESY spectrum of **1** in $CDCl_3$
Figure S11 ORTEP view of **1**
Figure S12 Crystal cell packing of **1**

Figure S13 IR spectrum of **2**
Figure S14 UV spectrum of **2**
Figure S15 (+)-HR-ESI-MS spectrum of **2**
Figure S16 ^{1}H NMR spectrum (400M) of **2** in CD_3OD
Figure S17 ^{13}C NMR spectrum (100M) of **2** in CD_3OD
Figure S18 DEPT 135 spectrum (100M) of **2** in CD_3OD
Figure S19 HSQC spectrum of **2** in CD_3OD
Figure S20 HMBC spectrum of **2** in CD_3OD
Figure S21 ^{1}H-^{1}H COSY spectrum of **2** in CD_3OD
Figure S22 NOESY spectrum of **2** in CD_3OD
Figure S23 Experimental and calculated ECD spectra of **2**

Figure S24 IR spectrum of **3**
Figure S25 UV spectrum of **3**
Figure S26 (+)-HR-ESI-MS spectrum of **3**
Figure S27 ^{1}H NMR spectrum (400M) of **3** in CD_3OD
Figure S28 ^{13}C NMR spectrum (100M) of **3** in CD_3OD
Figure S29 DEPT 135 spectrum (100M) of **3** in CD_3OD
Figure S30 HSQC spectrum of **3** in CD_3OD
Figure S31 HMBC spectrum of **3** in CD_3OD
Figure S32 ^{1}H-^{1}H COSY spectrum of **3** in CD_3OD
Figure S33 NOESY spectrum of **3** in CD_3OD
Figure S34 ORTEP view of **3**
Figure S35 Crystal cell packing of **3**

Figure S36 Pictures of the colony and the culture of *Chaetomium sp 88194*
Figure S37 ITS sequence of *Chaetomium sp 88194*

Figure S1. IR spectrum of **1**

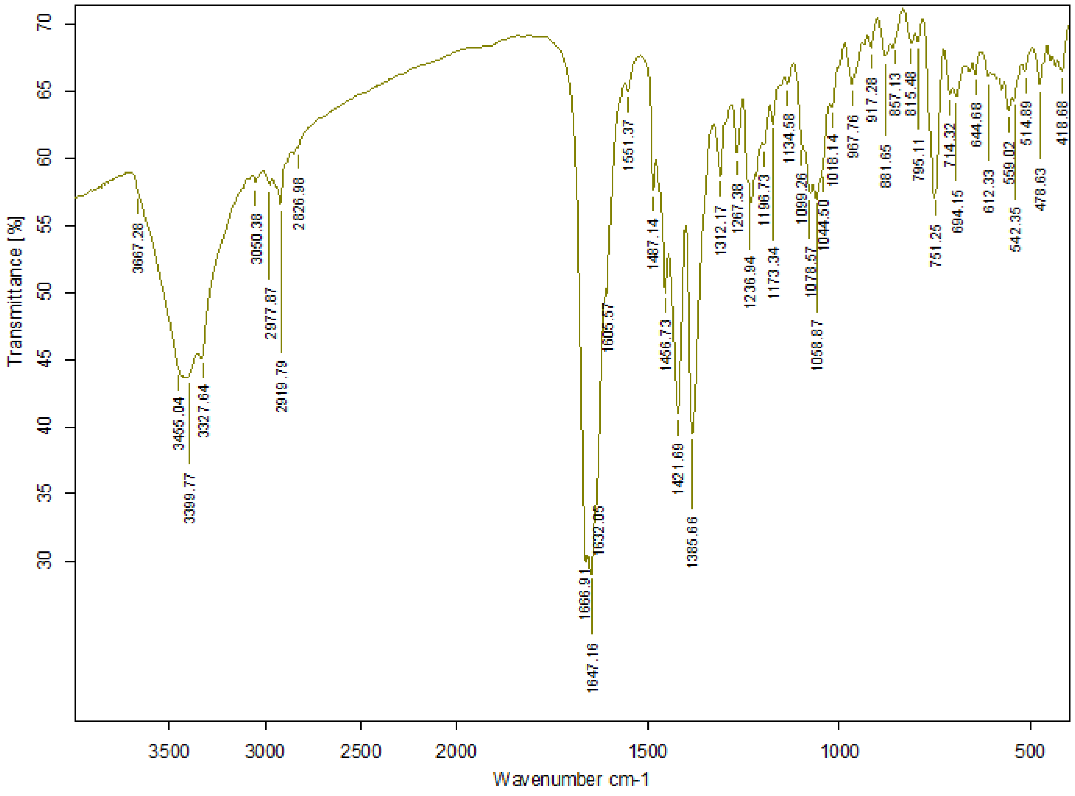

Figure S2 UV spectrum of **1**

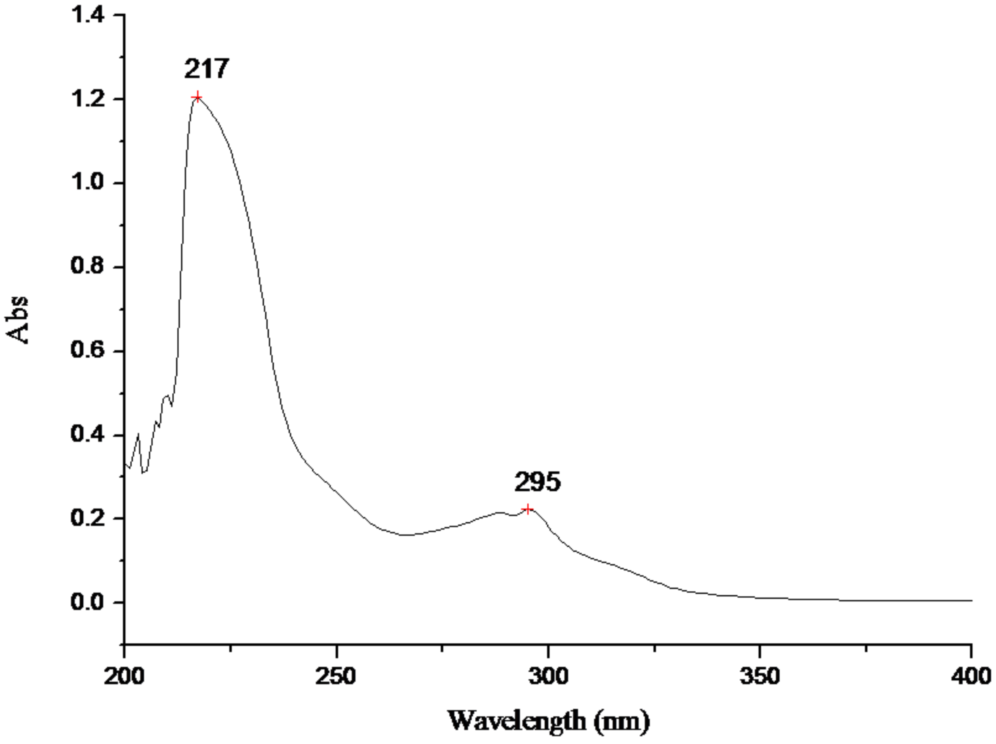

Figure S3 (+)-HR-ESI-MS spectrum of **1**

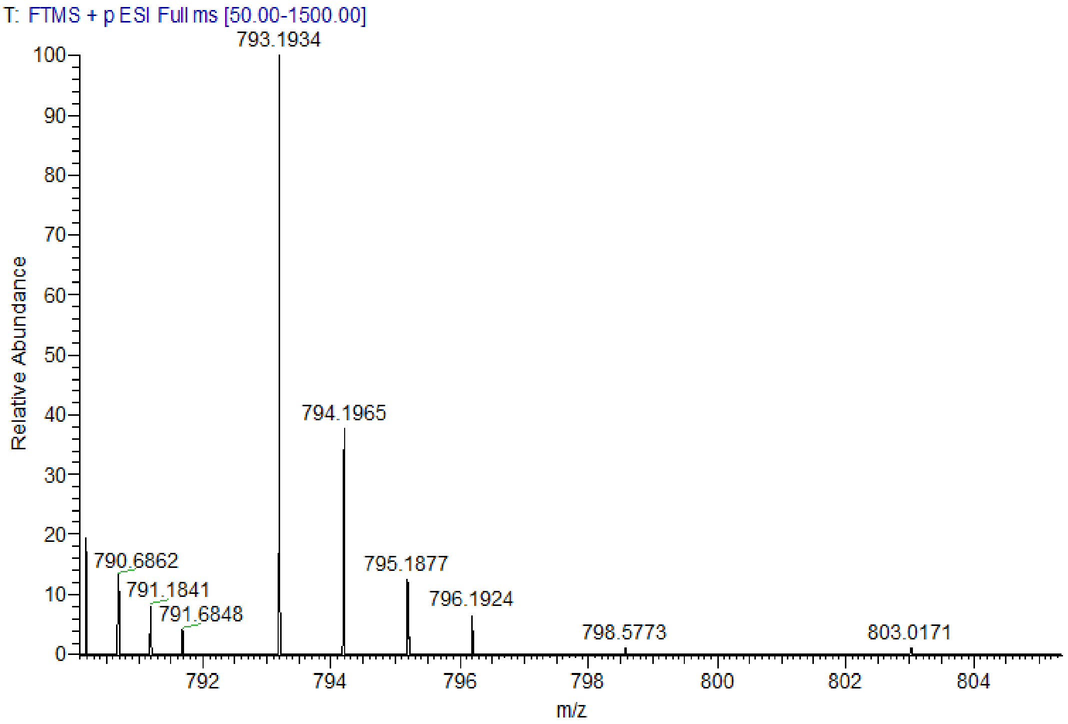

Figure S4 ^{1}H NMR spectrum (400M) of **1** in $CDCl_3$

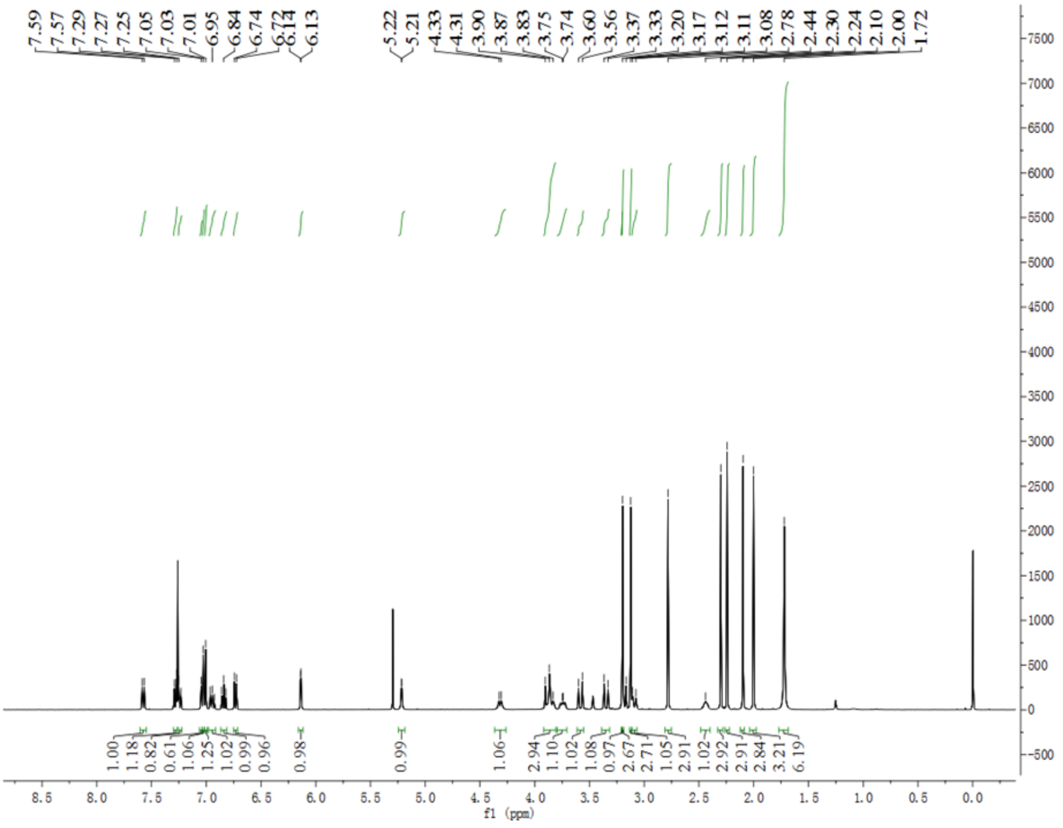

Figure S5 ^{13}C NMR spectrum (100M) of **1** in $CDCl_3$

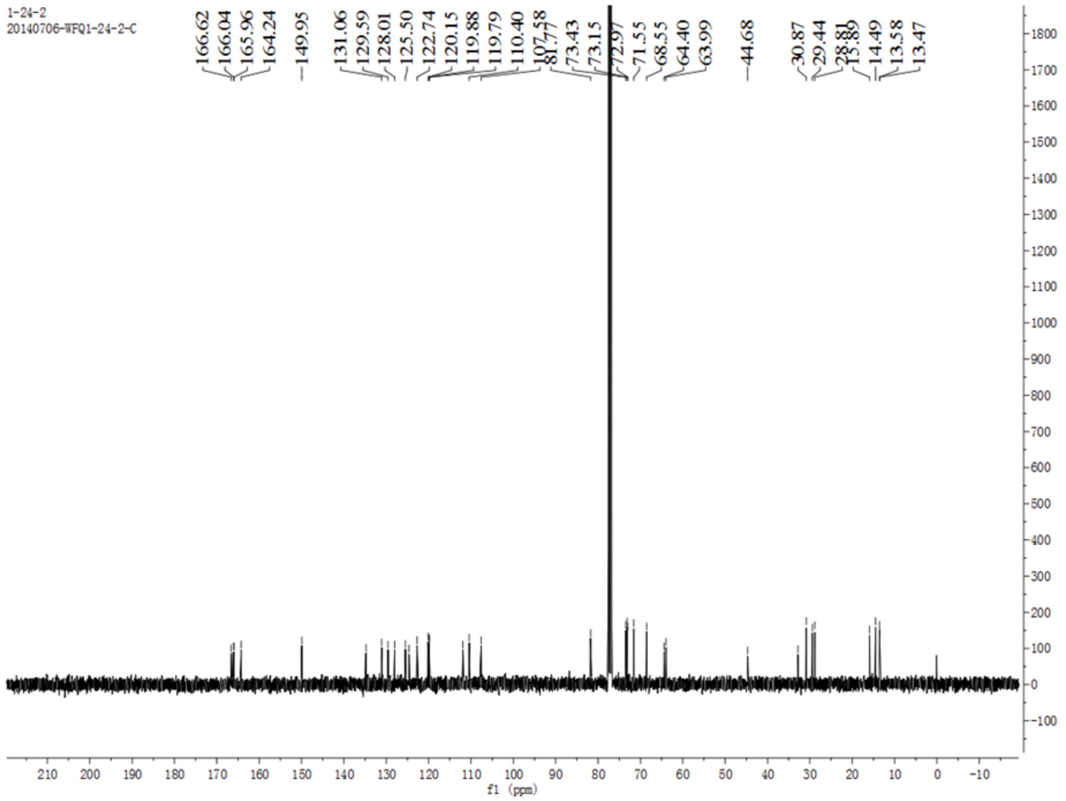

Figure S6 DEPT 135 spectrum (100M) of **1** in $CDCl_3$

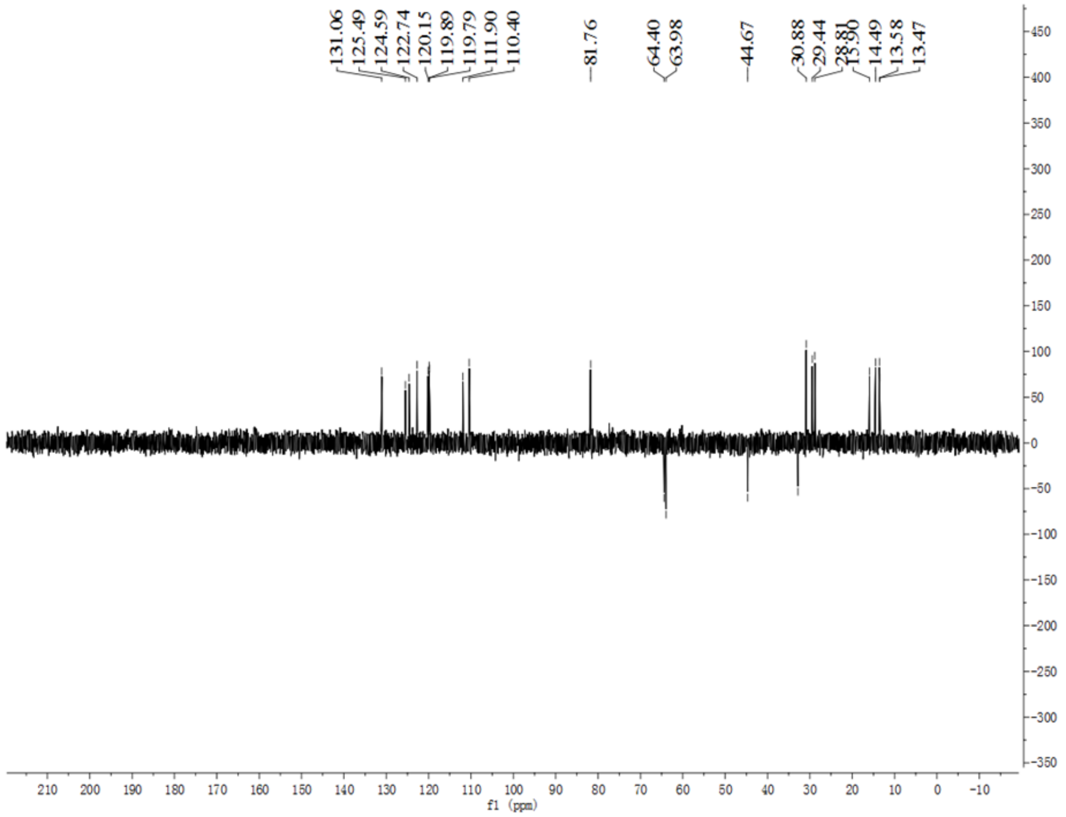

Figure S7 HSQC spectrum of **1** in $CDCl_3$

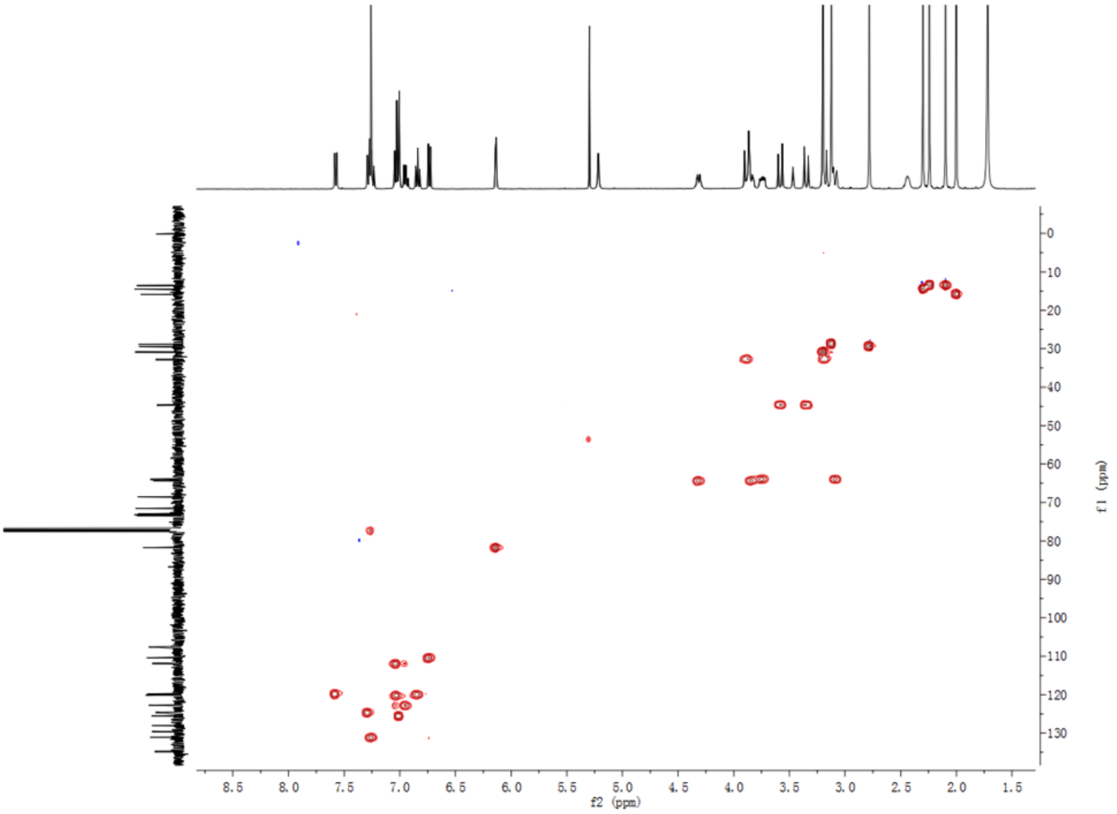

Figure S8 HMBC spectrum of **1** in $CDCl_3$

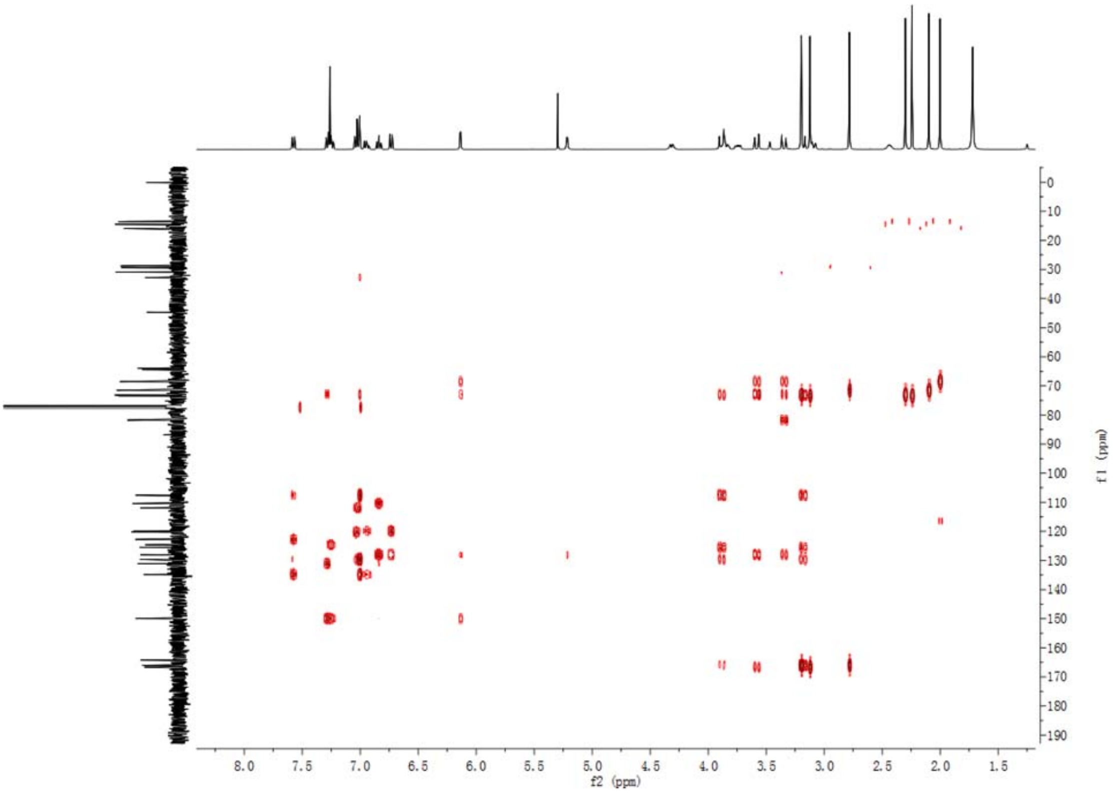

Figure S9 ^1H-^1H COSY spectrum of **1** in $CDCl_3$

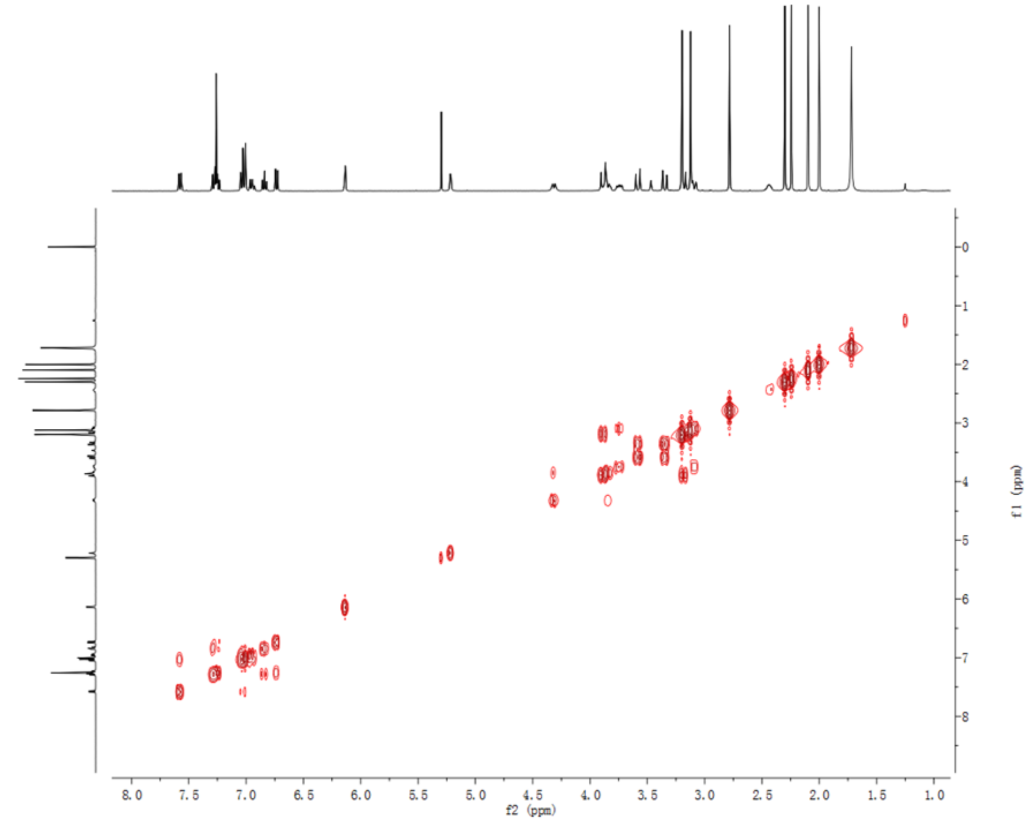

Figure S10 NOESY spectrum of **1** in $CDCl_3$

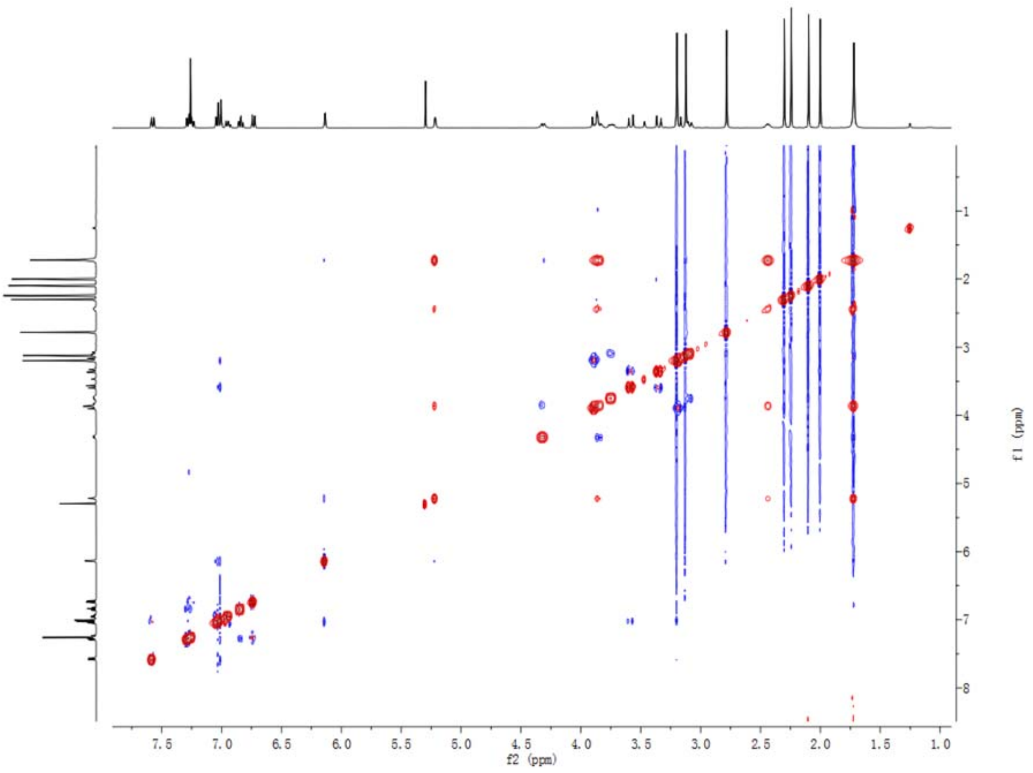

Figure S11 ORTEP View of **1**

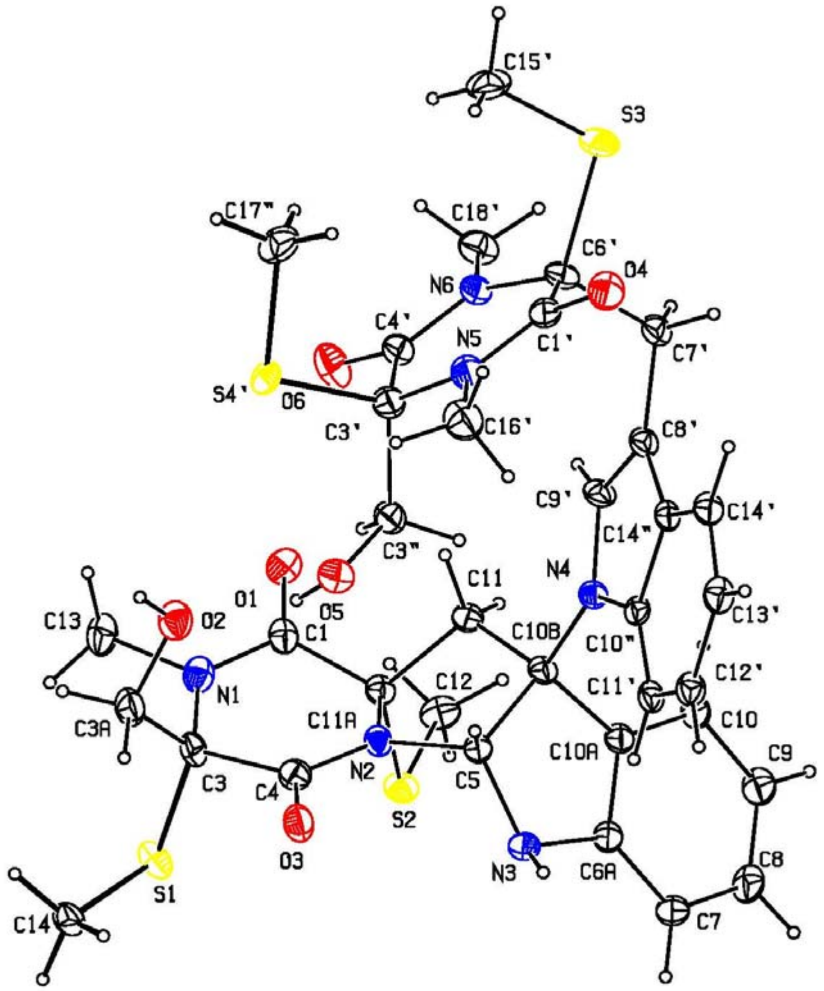

Figure S12 Crystal Cell packing of **1**

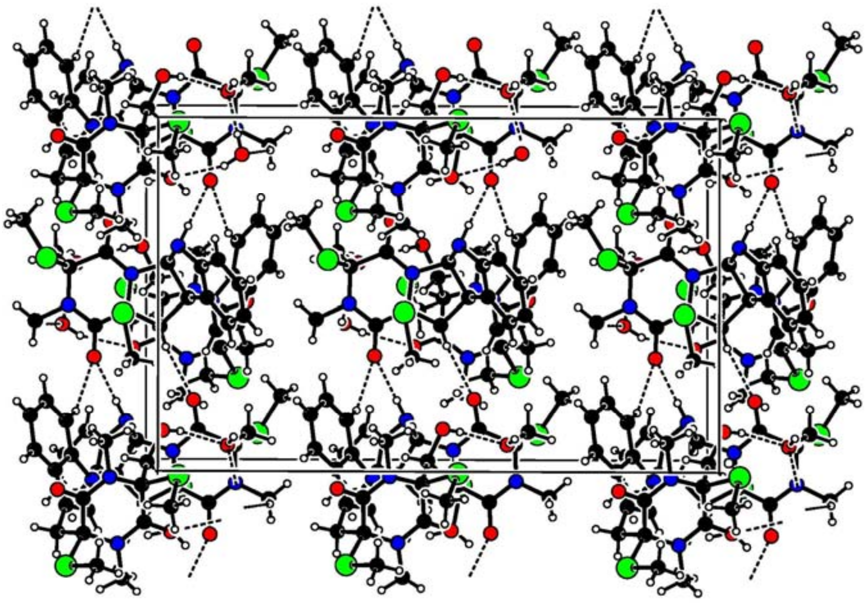

Figure S13 IR spectrum of **2**

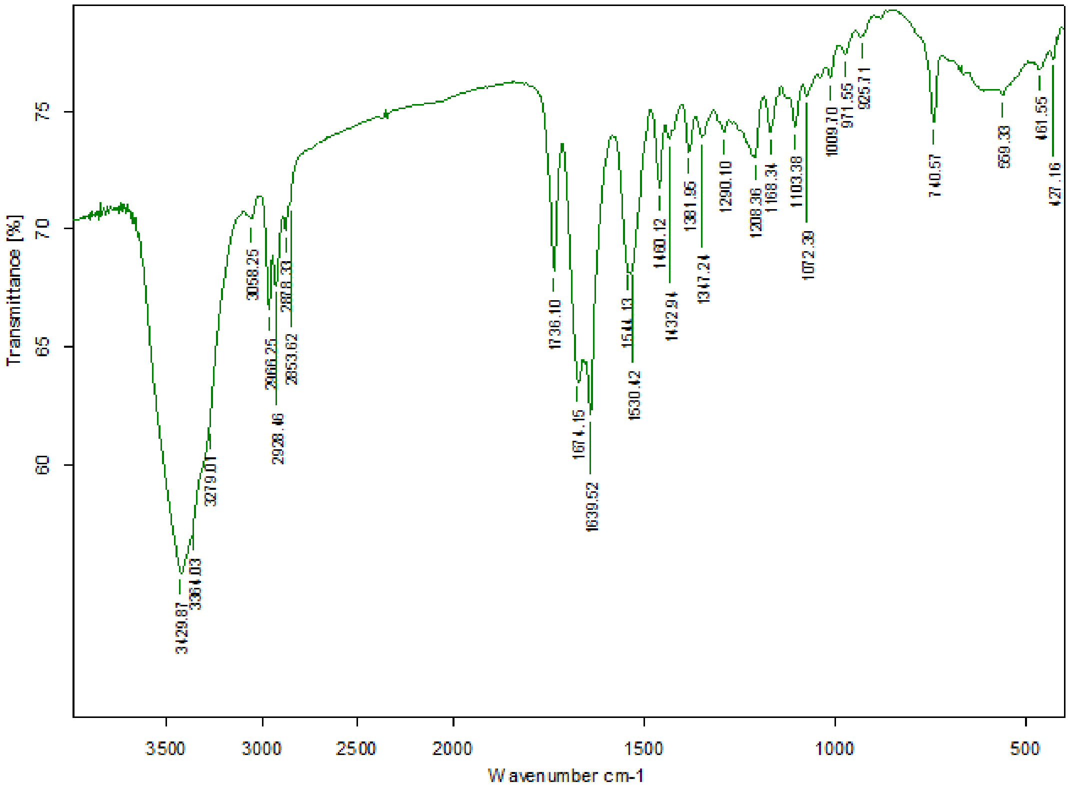

Figure S14 UV spectrum of **2**

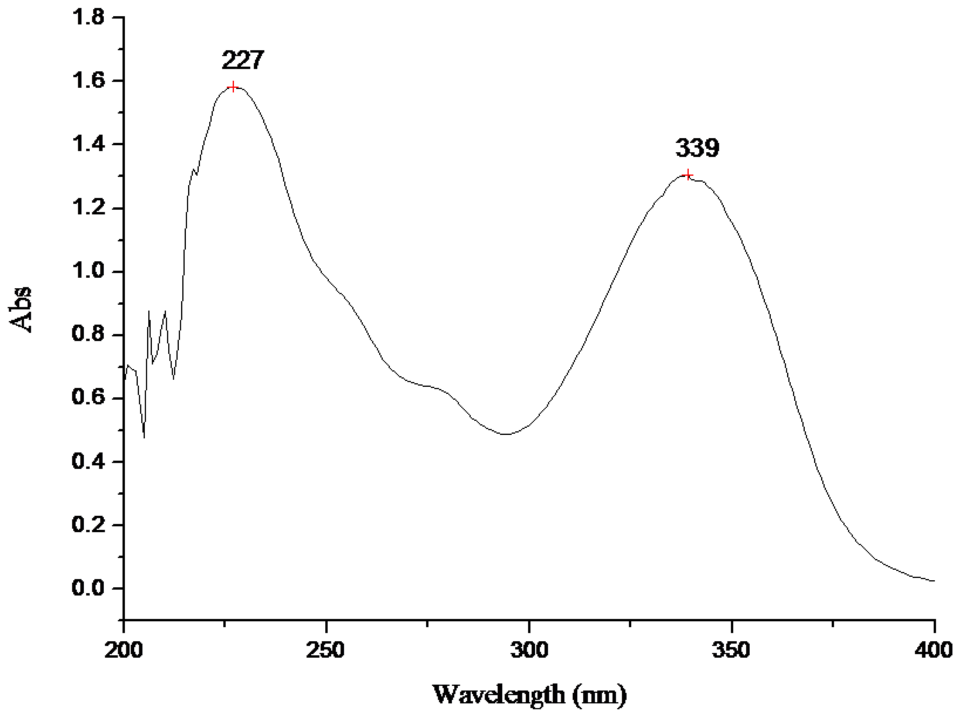

Figure S15 (+)-HR-ESI-MS spectrum of **2**

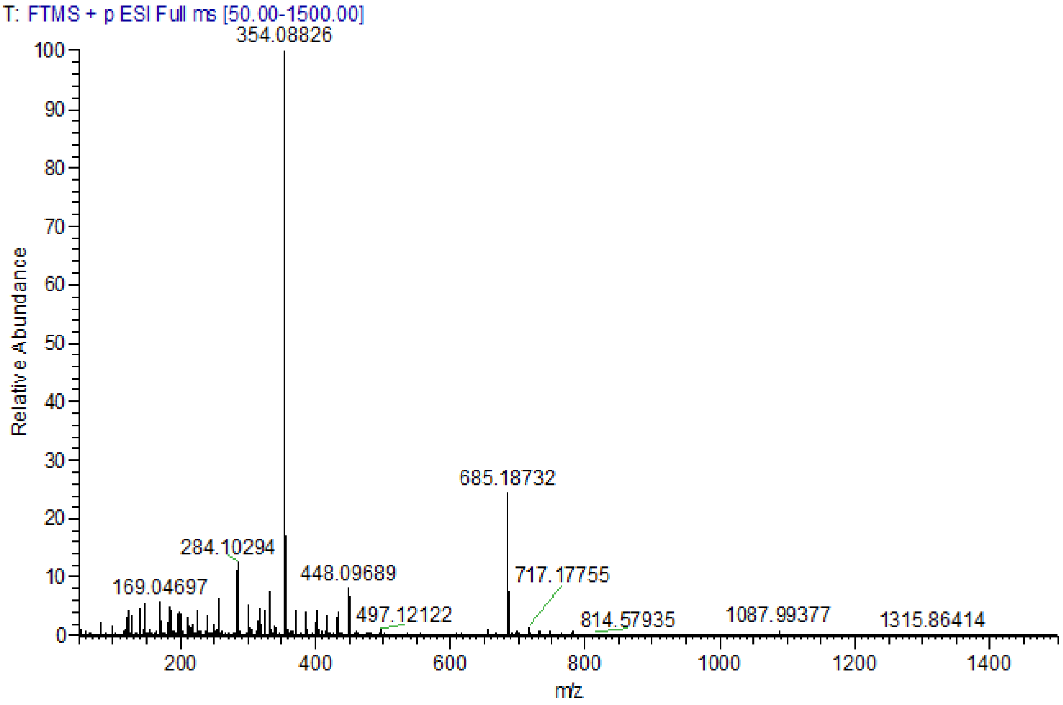

Figure S16 ^{1}H NMR spectrum (400M) of **2** in CD_3OD

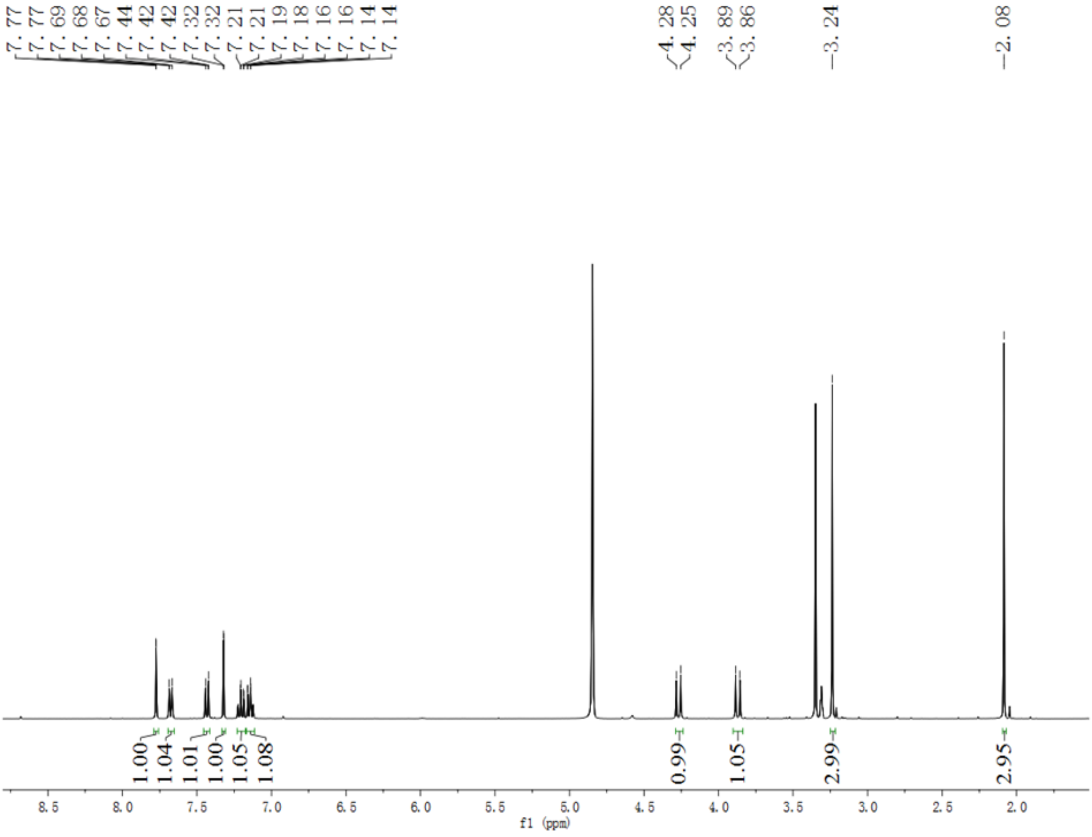

Figure S17 ^{13}C NMR spectrum (100M) of **2** in CD_3OD

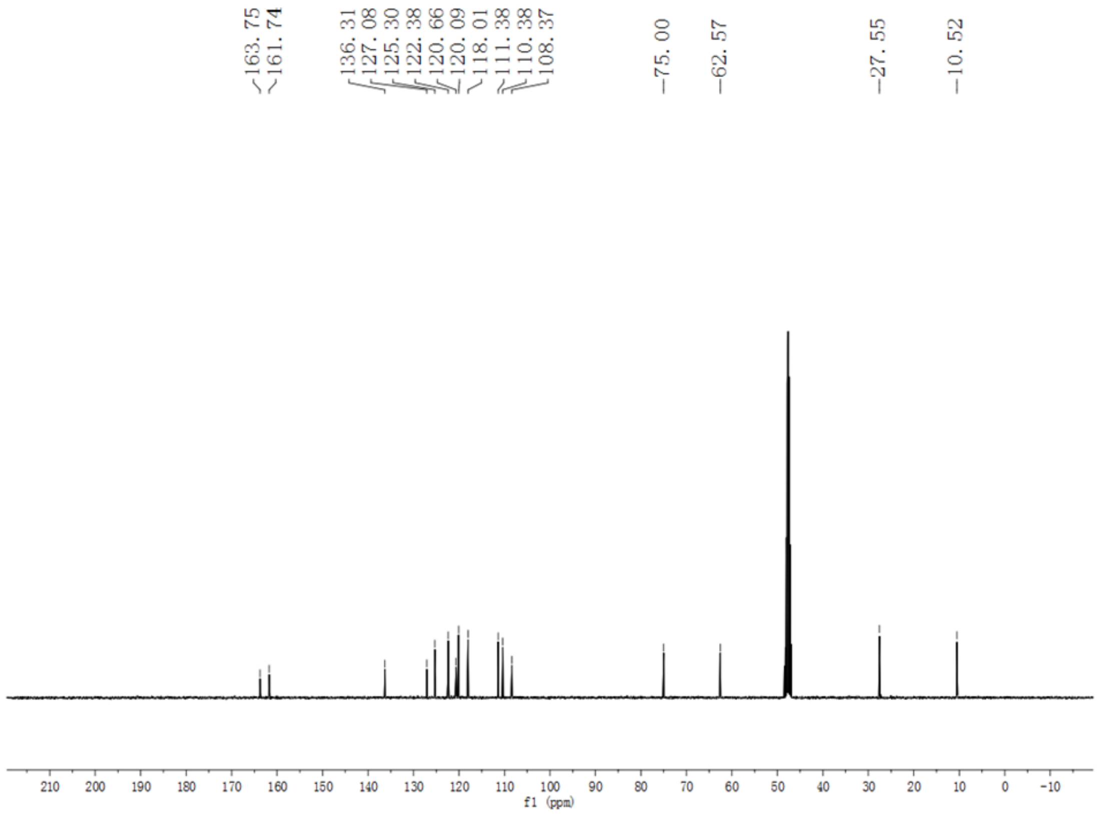

Figure S18 DEPT 135 spectrum (100M) of **2** in CD_3OD

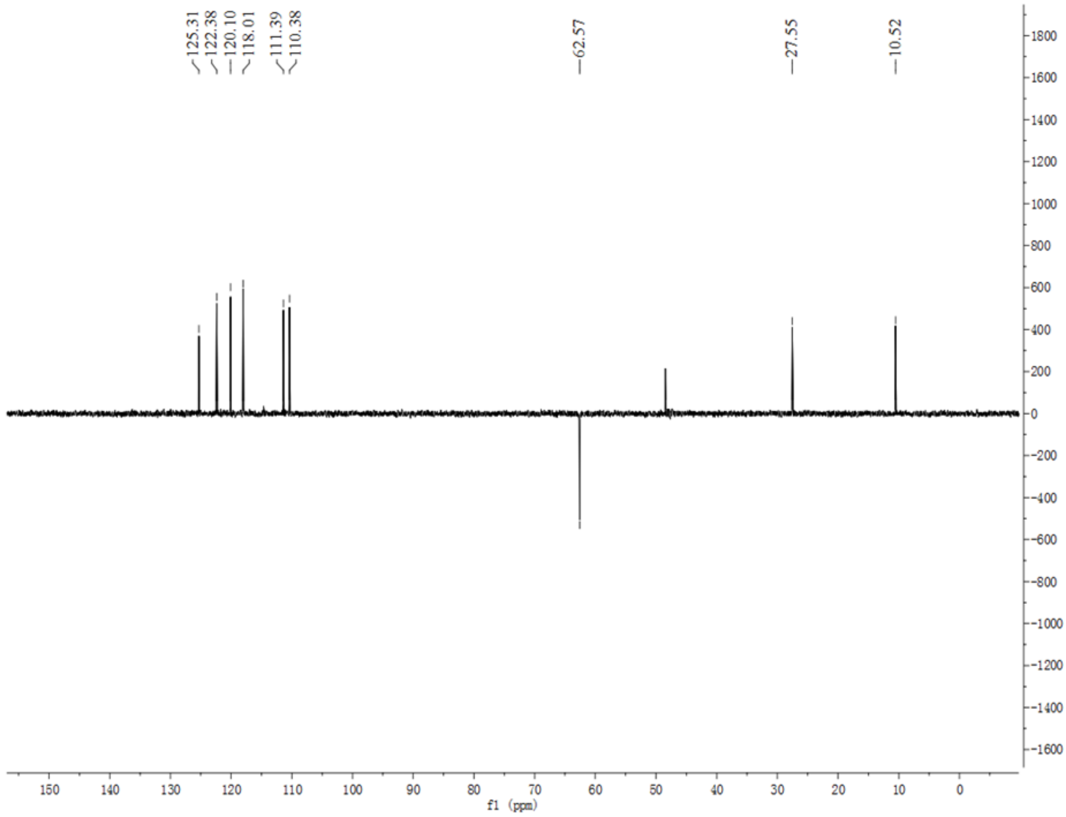

Figure S19 HSQC spectrum of **2** in CD_3OD

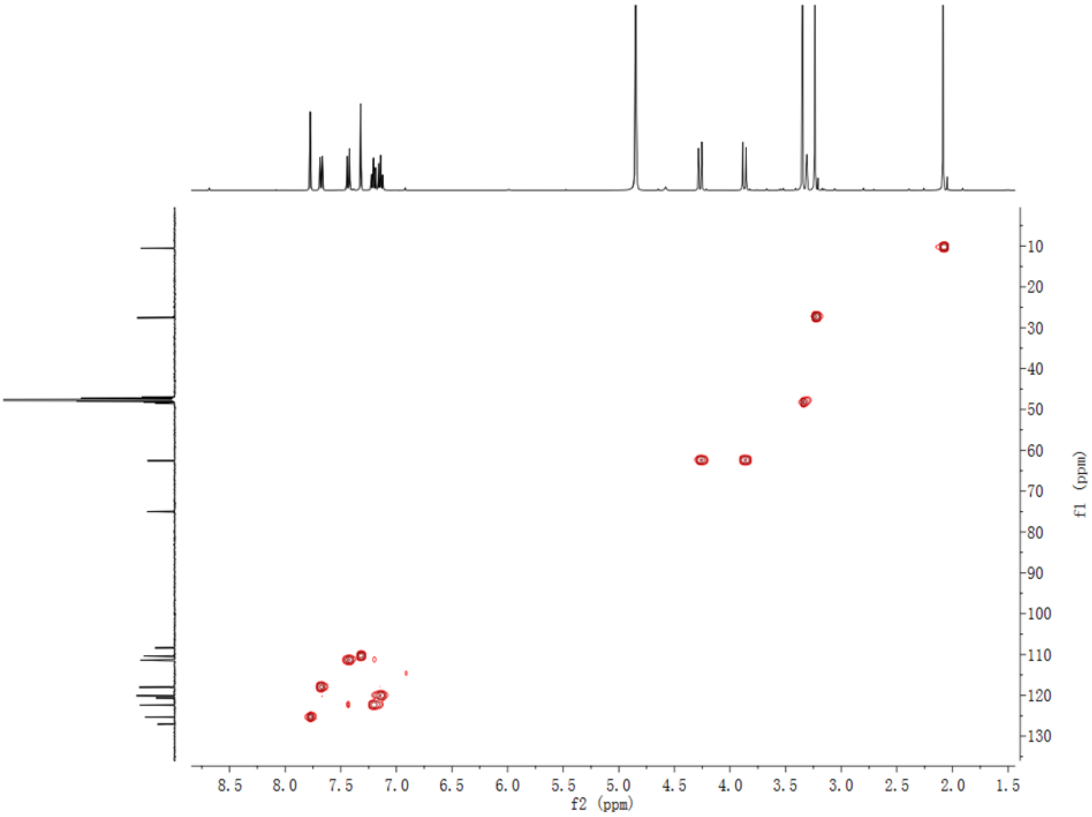

Figure S20 HMBC spectrum of **2** in CD_3OD

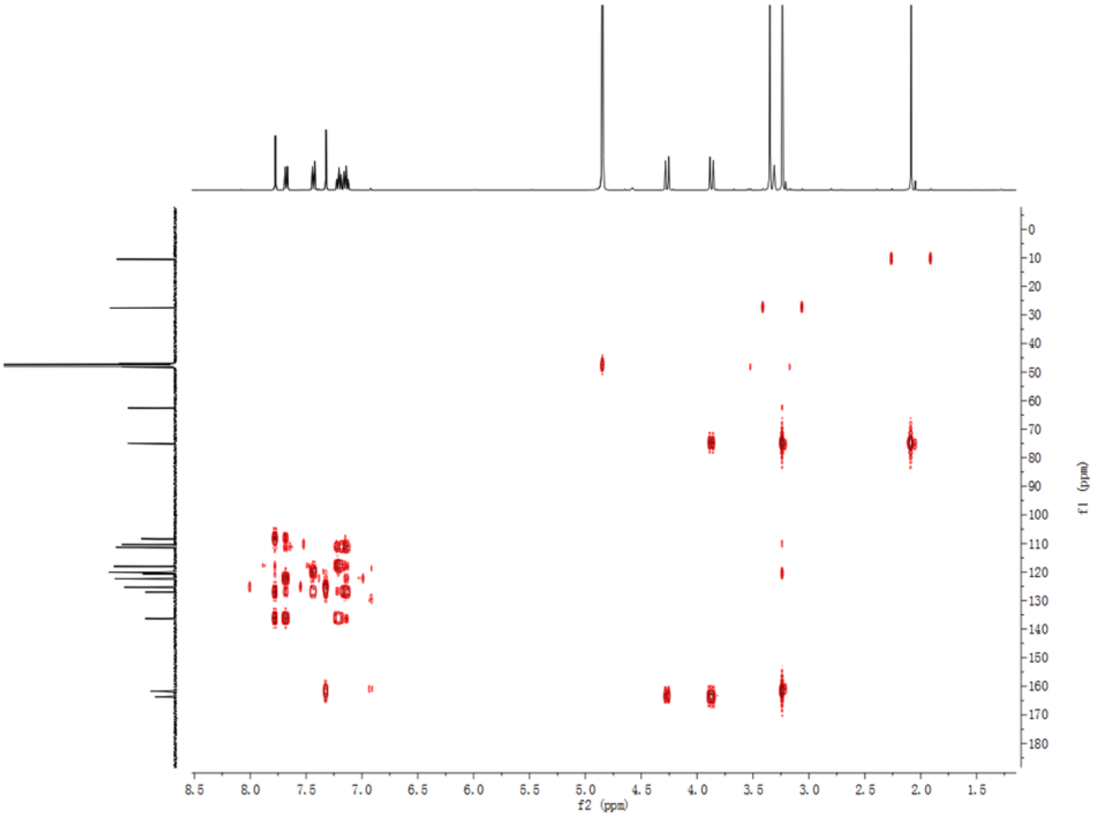

Figure S21 ^{1}H-^{1}H COSY spectrum of **2** in CD_3OD

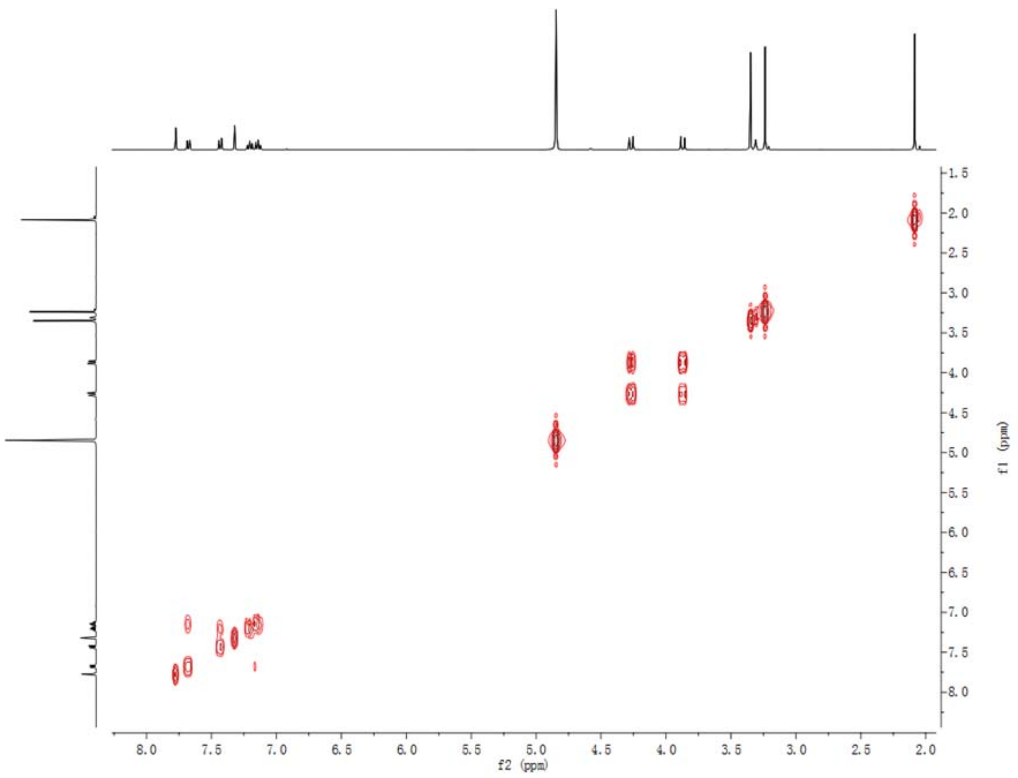

Figure S22 NOESY spectrum of **2** in CD_3OD

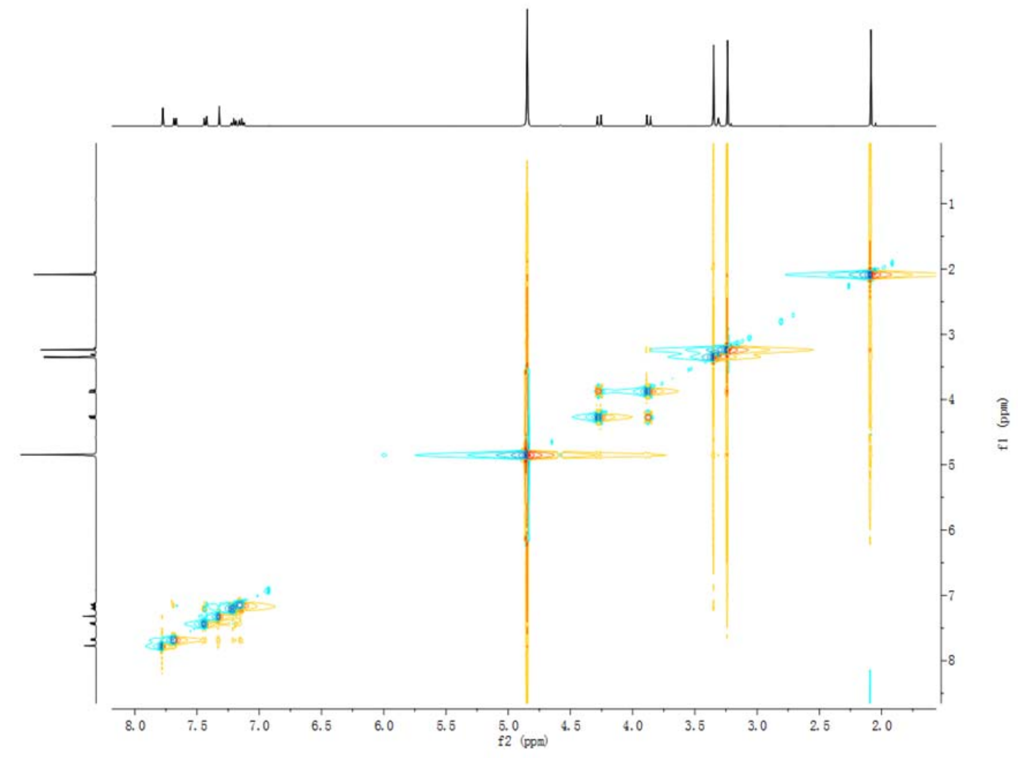

Figure S23 experimental and calculated ECD spectra of **2**

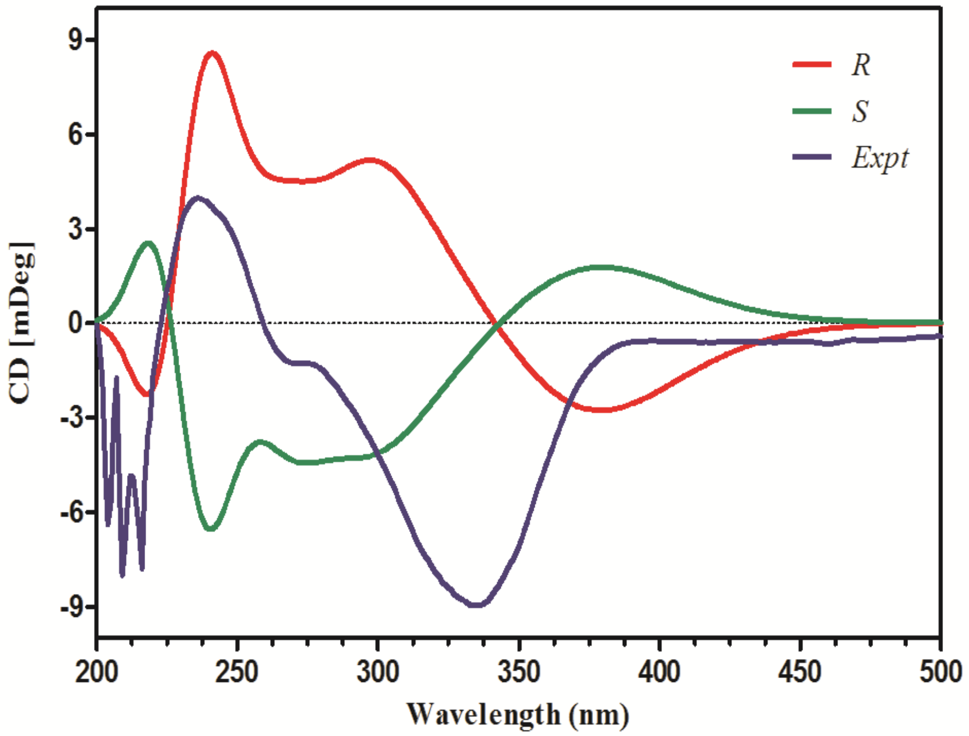

Figure S24 IR spectrum of **3**

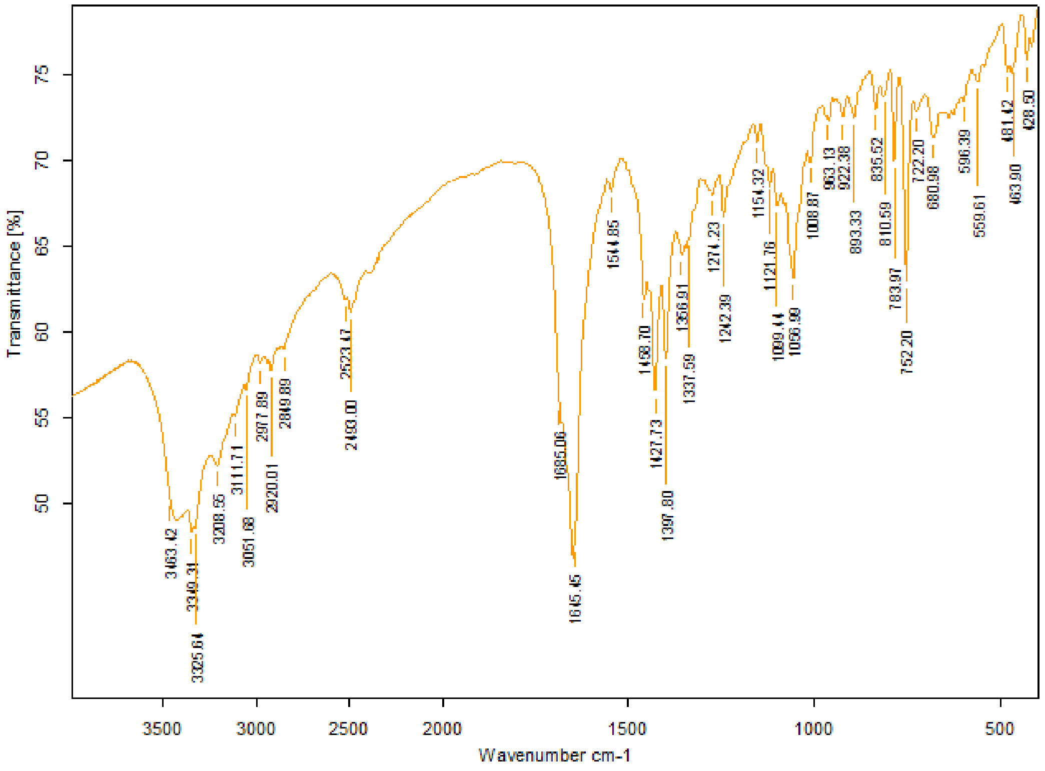

Figure S25 UV spectrum of **3**

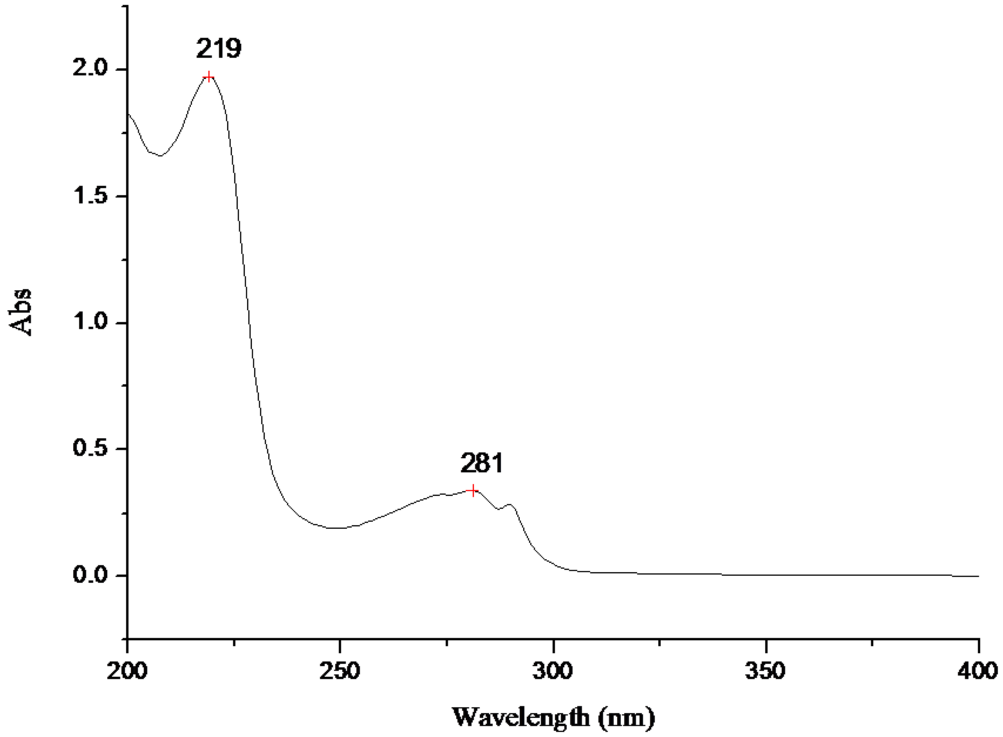

Figure S26 (+)-HR-ESI-MS spectrum of **3**

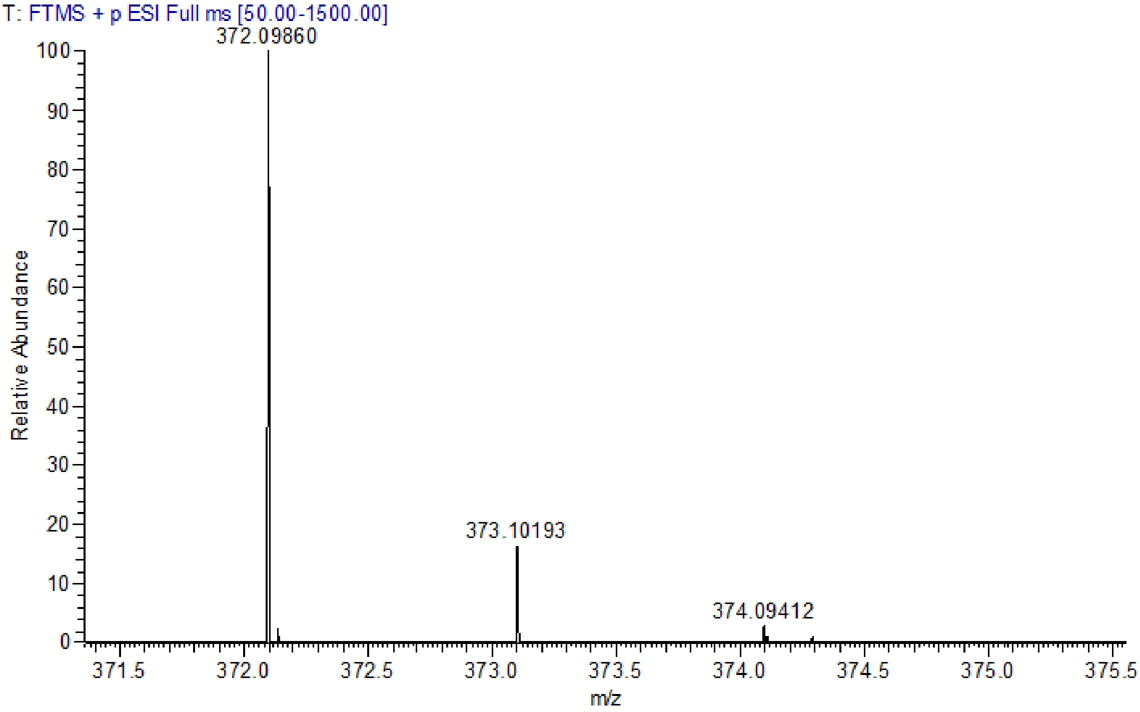

Figure S27 ^{1}H NMR spectrum (400M) of **3** in CD_3OD

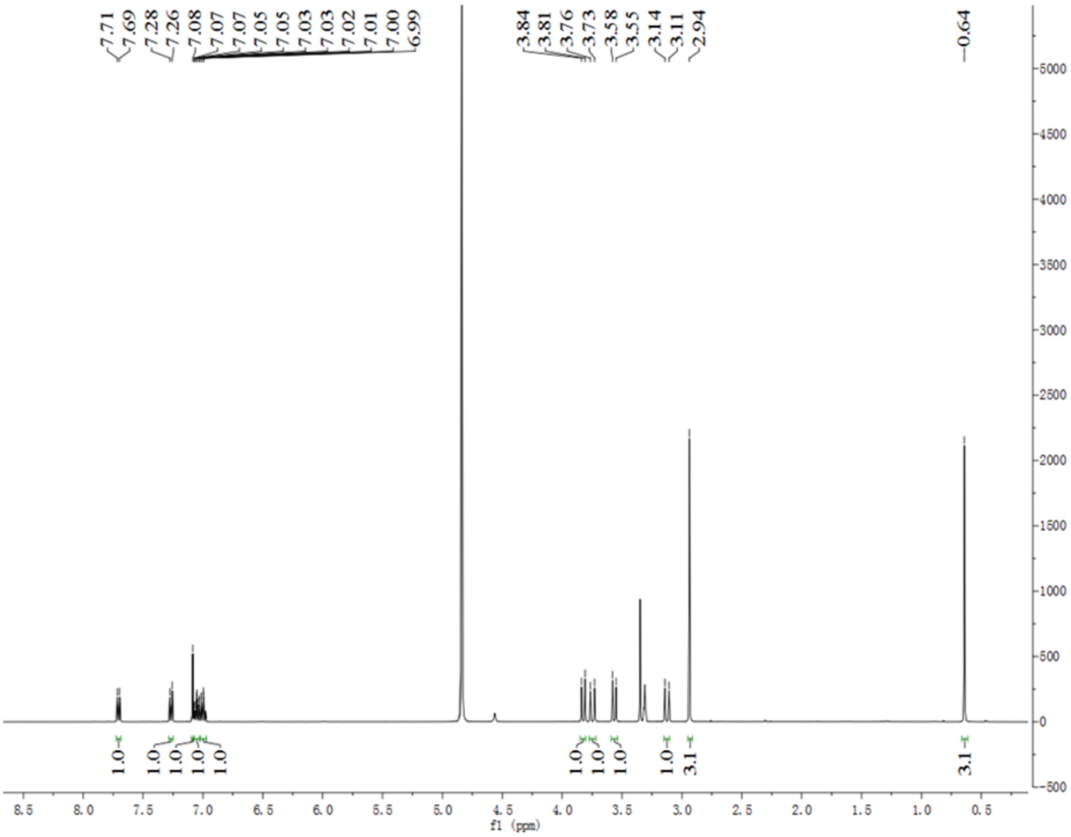

Figure S28 ^{13}C NMR spectrum (100M) of **3** in CD_3OD

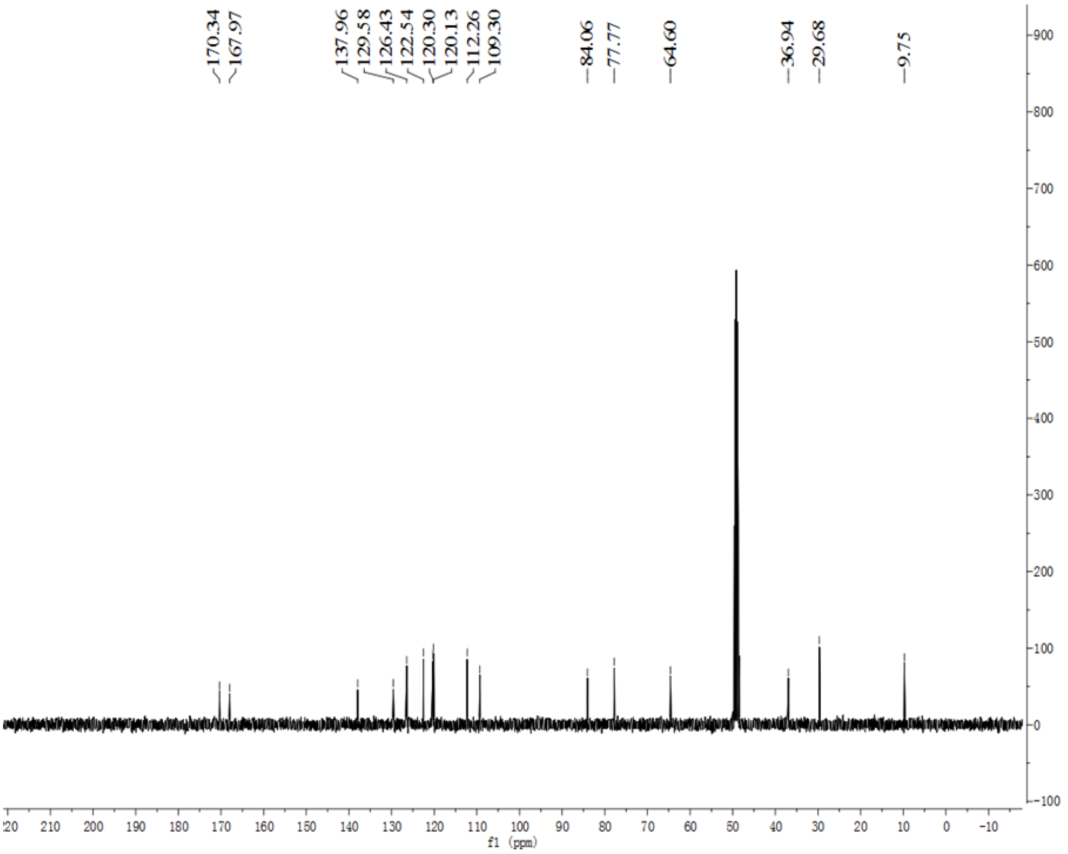

Figure S29 DEPT 135 spectrum (100M) of **3** in CD_3OD

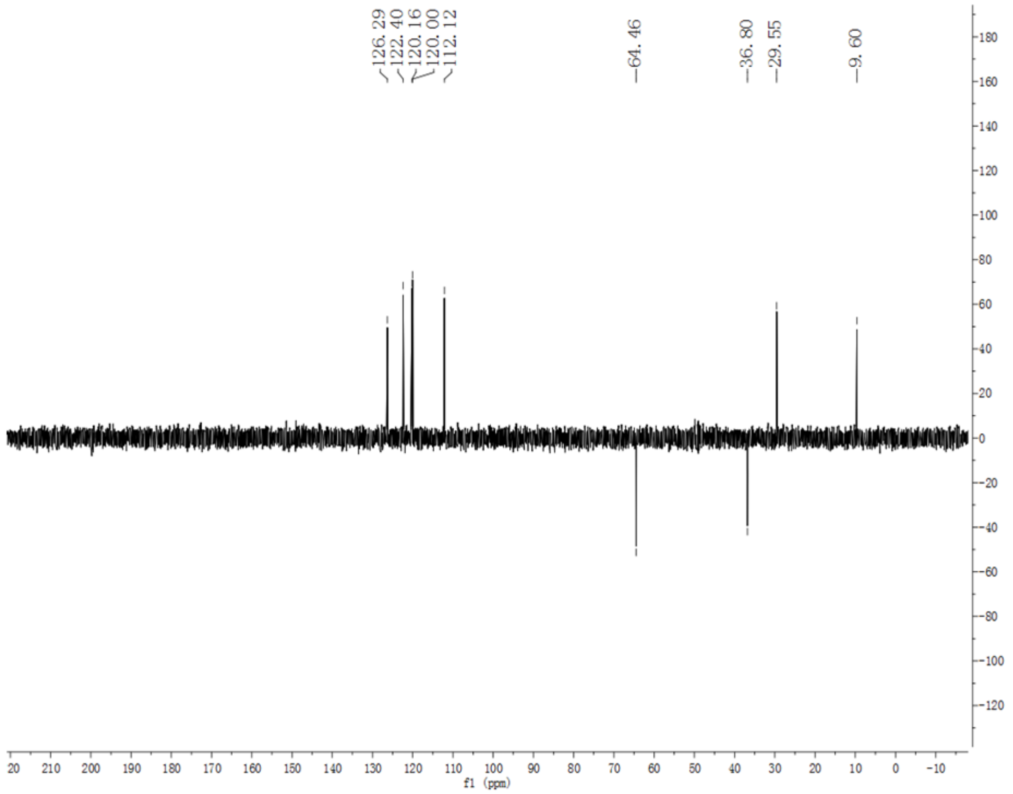

Figure S30 HSQC spectrum of **3** in CD_3OD

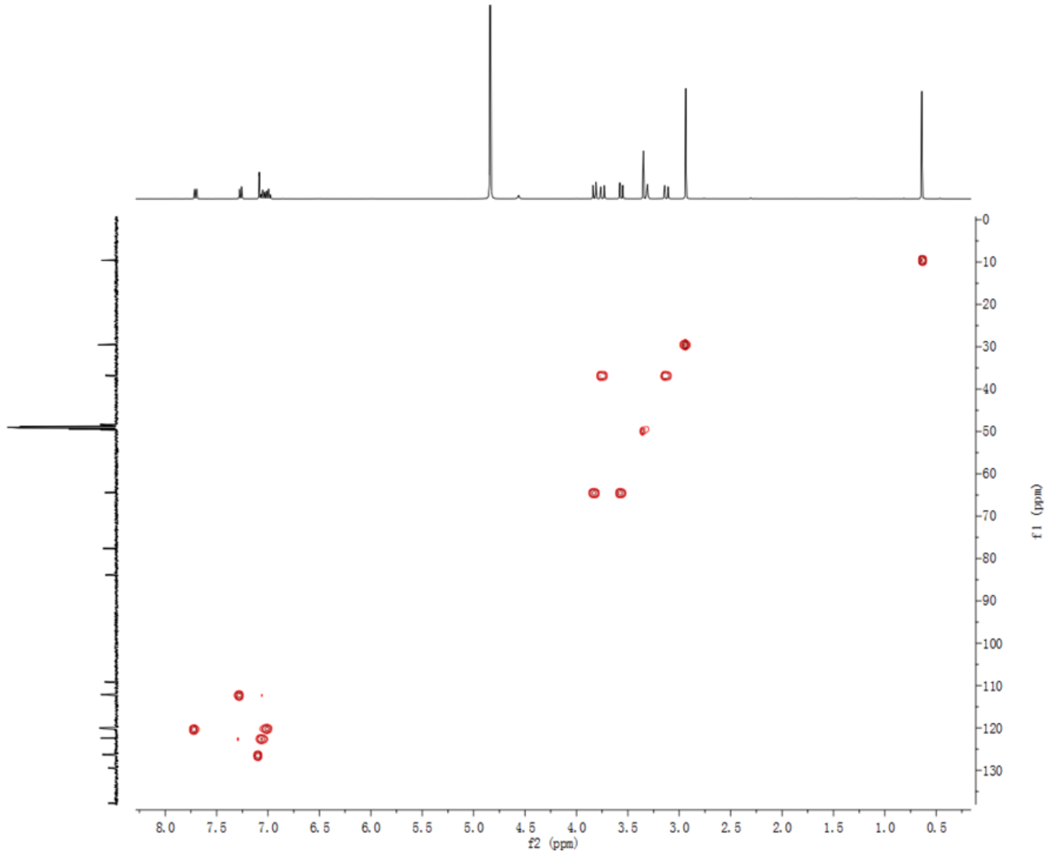

Figure S31 HMBC spectrum of **3** in CD_3OD

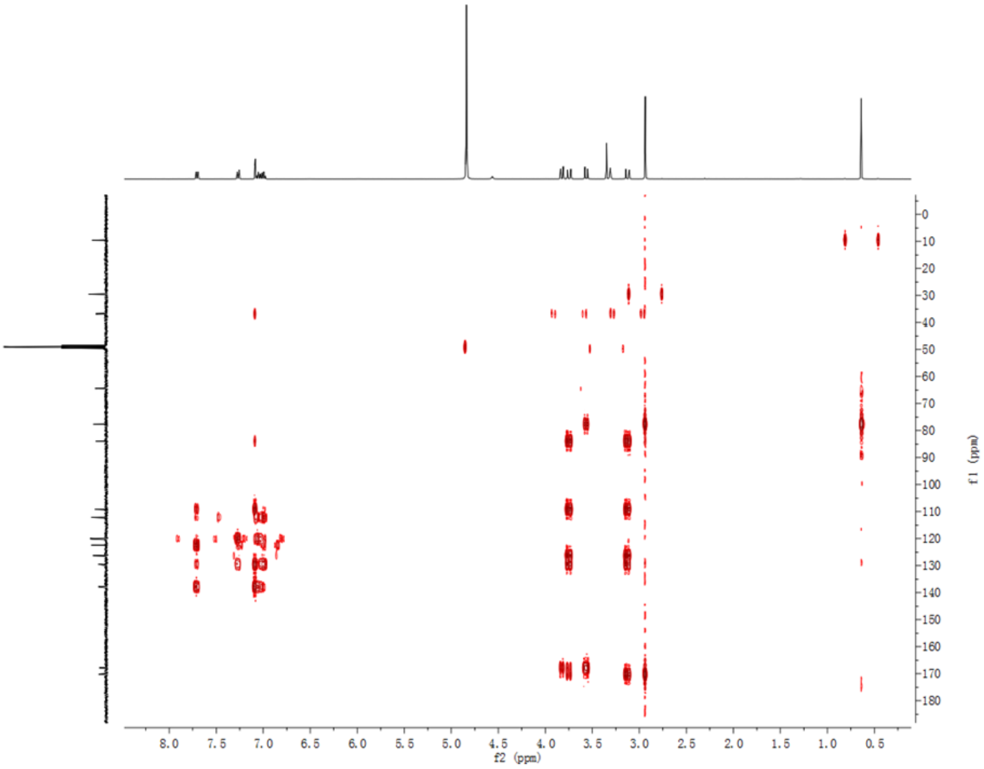

Figure S32 ^{1}H-^{1}H COSY spectrum of **3** in CD_3OD

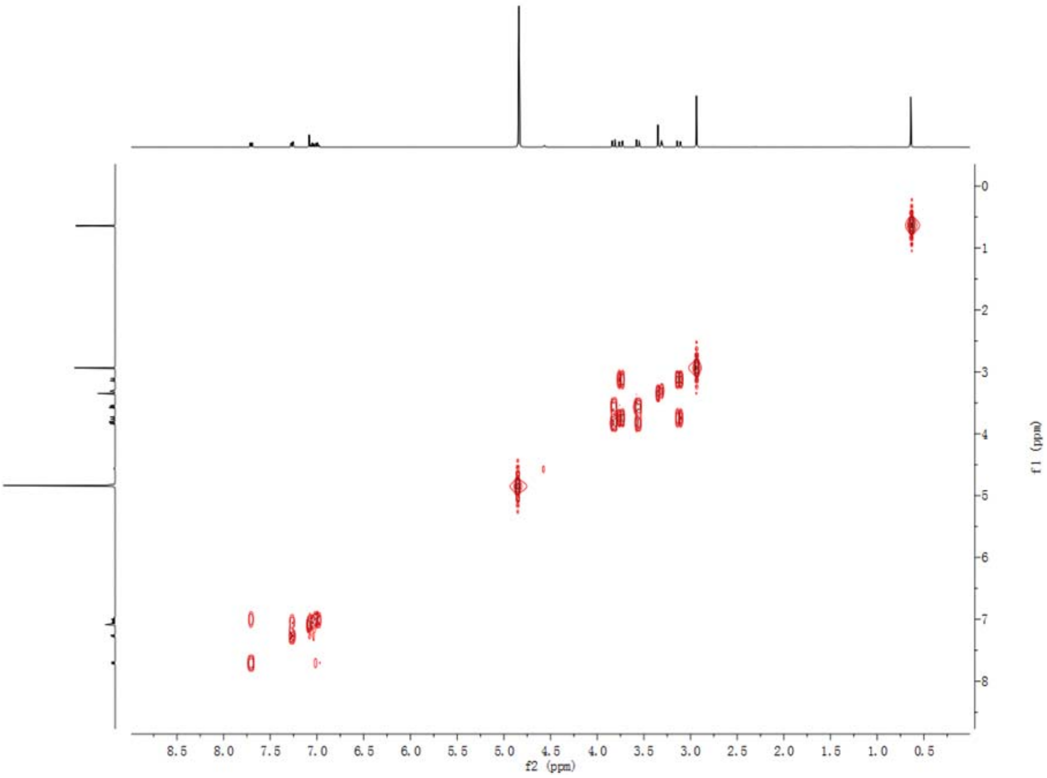

Figure S33 NOESY spectrum of **3** in CD_3OD

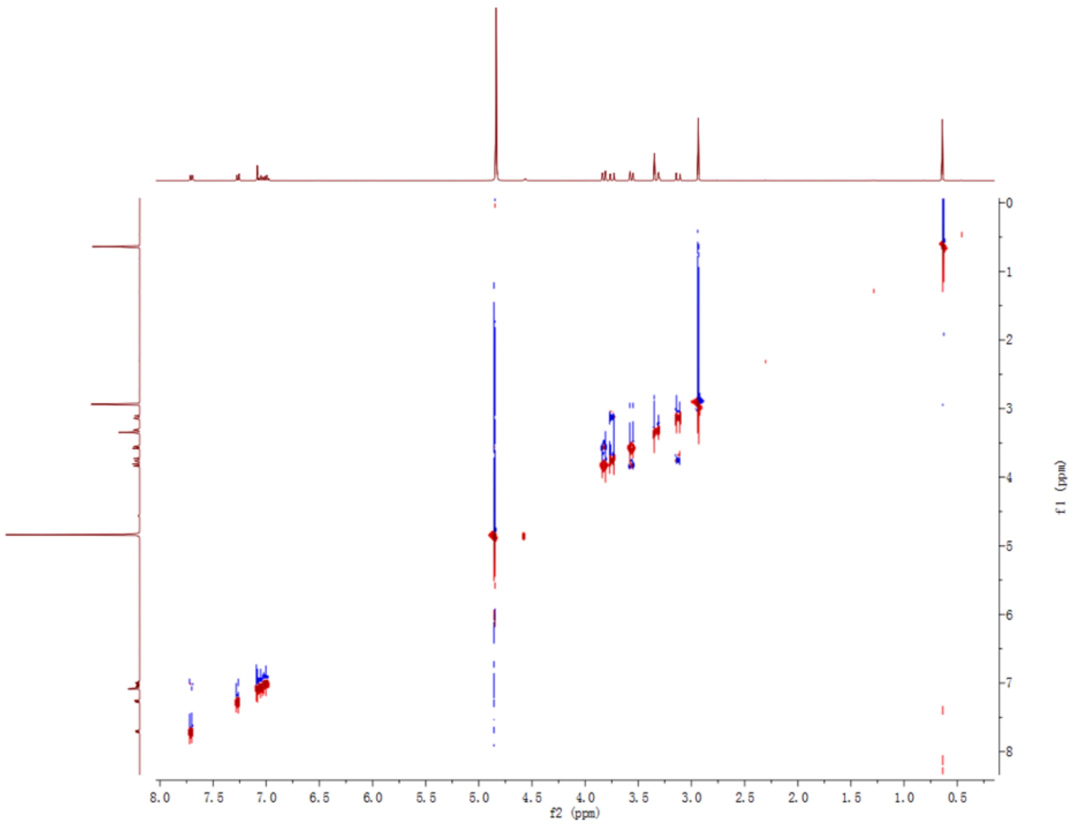

Figure S34 ORTEP View of **3**

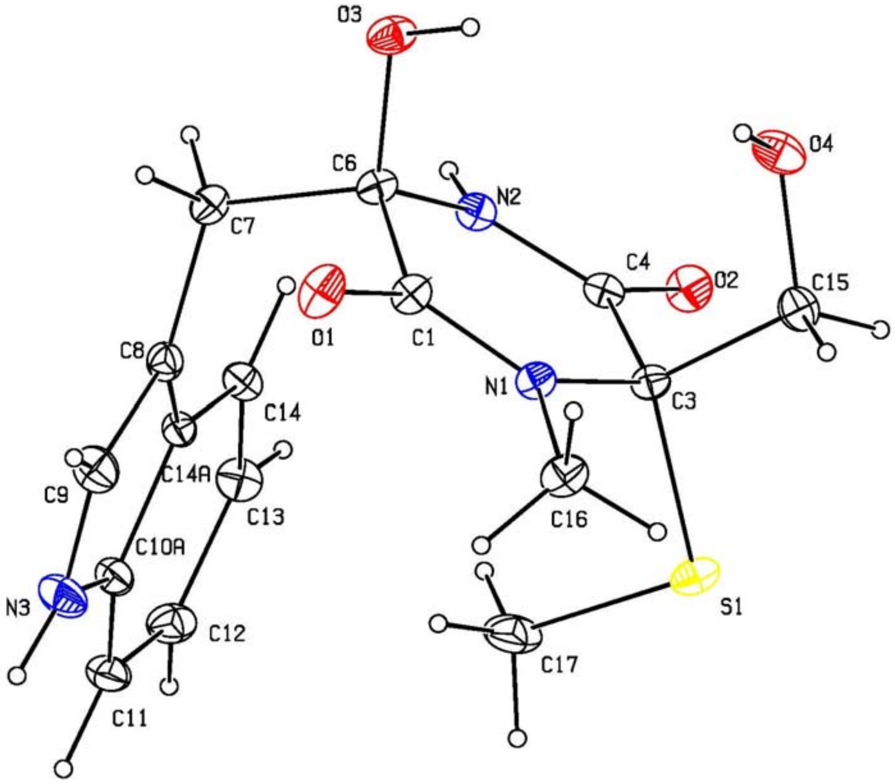

Figure S35 Crystal Cell packing of **3**

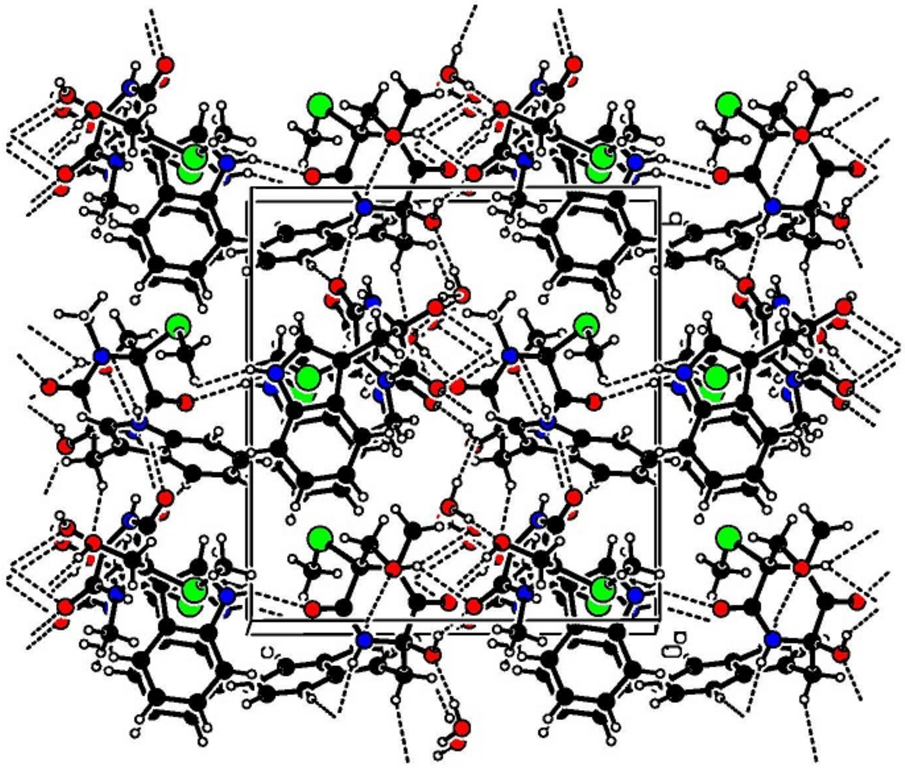

Figure S36 Pictures of the colony and the culture of *Chaetomium sp 88194*

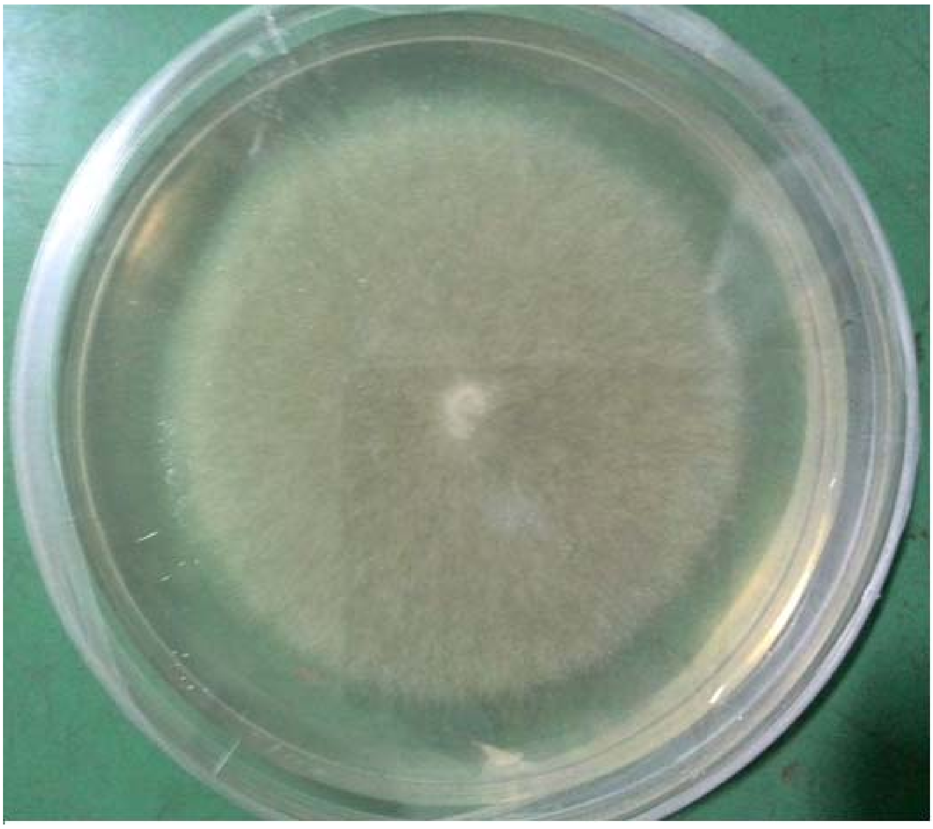

Figure S37 ITS sequence of *Chaetomium sp 88194*

Primers are marked; ITS1 and ITS4 were used
GTAATTCCGTAGGGGTGACCTGCGGAGGGATCATTACAGAGTTGCAAAACTC
CCTAAACCATTGTGAACGTTACCTAAACCGTTGCTTCGGCGGGCGGCGCCGG
GGTTTACCCCCCGGGCGCCCCTGGGCCCCACCGCGGGCGCCCGCCGGAGGTC
ACCAAACTCTTGATAATTTATGGCCTCTCTGAGTCTTCTGTACTGAATAAGTC
AAAACTTTCAACAACGGATCTCTTGGTTCTGGCATCGATGAAGAACGCAGCG
AAATGCGATAAGTAATGTGAATTGCAGAATTCAGTGAATCATCGAATCTTTG
AACGCACATTGCGCCCGCCAGTATTCTGGCGGGCATGCCTGTTCGAGCGTCAT
TTCAACCATCAAGCCCCGGGCTTGTGTTGGGGACCTGCGGCTGCCGCAGGCC
CTGAAAAGCAATGGCGGGCTCGCTGTCACACCGAGCGTAATAACATACATCT
CGCTCTGGGCGTGCTGCGGGTTCCGGCCGTTAAACCACCTTTTAACCCAAGGT
TGACCTCGGATCAAGTAGGAAGACCCGCTGAACTTAAGCATATCAATAAACC
AGAAGAAA
