# Supplementary material for: Indole diketopiperazines from endophytic Chaetomium sp 88194 induce breast cancer cell apoptotic death
Source: Sci Rep. 2015 Mar 19;5:9294. doi: 10.1038/srep09294 (PMC4365412; doi:10.1038/srep09294)

checkCIF/PLATON report

Structure factors have been supplied for datablock(s) g__140718d_work_cu_140718d_0m

THIS REPORT IS FOR GUIDANCE ONLY. IF USED AS PART OF A REVIEW PROCEDURE FOR PUBLICATION, IT SHOULD NOT REPLACE THE EXPERTISE OF AN EXPERIENCED CRYSTALLOGRAPHIC REFEREE.

No syntax errors found. CIF dictionary Interpreting this report

Datablock: g__140718d_work_cu_140718d_0m

```
Bond precision:  C-C = 0.0056 A          Wavelength=1.54178

Cell:            a=13.3191(3)     b=13.7927(3)       c=21.8874(5)
                 alpha=90         beta=96.045(1)     gamma=90
Temperature:     296 K
```

	Calculated	Reported
Volume	3998.50(16)	3998.50(15)
Space group	P 21	P 21
Hall group	P 2yb	P 2yb
Moiety formula	C35 H42 N6 O6 S4, H2 O	C35 H44 N6 O7 S4
Sum formula	C35 H44 N6 O7 S4	C35 H44 N6 O7 S4
Mr	789.00	789.00
Dx,g cm-3	1.311	1.311
Z	4	4
Mu (mm-1)	2.623	2.623
F000	1664.0	1664.0
F000’	1673.76	
h,k,lmax	14,15,24	14,14,24
Nref	11302[5929]	10529
Tmin,Tmax	0.621,0.769	0.622,0.779
Tmin’	0.564	

```
Correction method= MULTI-SCAN

Data completeness= 1.78/0.93      Theta(max)= 58.420

R(reflections)= 0.0437( 7411)     wR2(reflections)= 0.1312( 10529)

S = 1.067                    Npar= 1029
```

The following ALERTS were generated. Each ALERT has the format
test-name_ALERT_alert-type_alert-level.
Click on the hyperlinks for more details of the test.

Alert level A

PLAT415_ALERT_2_A Short Inter D-H..H-X H13A .. H2W1 .. 1.83 Ang.

Alert level B

THETM01_ALERT_3_B The value of sine(theta_max)/wavelength is less than 0.575
Calculated sin(theta_max)/wavelength = 0.5525
PLAT035_ALERT_1_B No _chemical_absolute_configuration info given . Please Do !
PLAT112_ALERT_2_B ADDSYM Detects Additional (Pseudo) Symm. Elem... Z Check
PLAT414_ALERT_2_B Short Intra D-H..H-X H3"1 .. H5A .. 1.81 Ang.
PLAT415_ALERT_2_B Short Inter D-H..H-X H8 .. H15E .. 2.06 Ang.
PLAT417_ALERT_2_B Short Inter D-H..H-D H8 .. H1W1 .. 2.00 Ang.
PLAT420_ALERT_2_B D-H Without Acceptor O2 - H2 ... Please Check
PLAT420_ALERT_2_B D-H Without Acceptor O8 - H8 ... Please Check

---

Alert level C

PLAT029_ALERT_3_C _diffrn_measured_fraction_theta_full Low .... 0.973 Note
PLAT089_ALERT_3_C Poor Data / Parameter Ratio (Zmax < 18) .... 5.76 Note
PLAT220_ALERT_2_C Large Non-Solvent C Ueq(max)/Ueq(min) Range 4.2 Ratio
PLAT222_ALERT_3_C Large Non-Solvent H Uiso(max)/Uiso(min) .. 4.9 Ratio
PLAT230_ALERT_2_C Hirshfeld Test Diff for N2 -- C4 .. 6.7 su
PLAT230_ALERT_2_C Hirshfeld Test Diff for C1' -- C6' .. 6.5 su
PLAT234_ALERT_4_C Large Hirshfeld Difference S4' -- C17" .. 0.17 Ang.
PLAT242_ALERT_2_C Low Ueq as Compared to Neighbors for ..... C3' Check
PLAT242_ALERT_2_C Low Ueq as Compared to Neighbors for ..... S8 Check
PLAT340_ALERT_3_C Low Bond Precision on C-C Bonds .... 0.0056 Ang.
PLAT414_ALERT_2_C Short Intra D-H..H-X H11 .. H3E1 .. 1.90 Ang.
PLAT415_ALERT_2_C Short Inter D-H..H-X H2 .. H15B .. 2.11 Ang.
PLAT480_ALERT_4_C Long H...A H-Bond Reported H2W2 .. S4 .. 2.94 Ang.

Alert level G

PLAT002_ALERT_2_G Number of Distance or Angle Restraints on AtSite 29 Note
PLAT003_ALERT_2_G Number of Uiso or Uij Restrained non-H Atoms ... 4 Report
PLAT005_ALERT_5_G No _iucr_refine_instructions_details in the CIF Please Do !
PLAT007_ALERT_5_G Number of Unrefined Donor-H Atoms .... 4 Report
PLAT042_ALERT_1_G Calc. and Reported MoietyFormula Strings Differ Please Check
PLAT230_ALERT_2_G Hirshfeld Test Diff for S4' -- C3' .. 7.5 su
PLAT230_ALERT_2_G Hirshfeld Test Diff for S4 -- C3' .. 8.9 su
PLAT230_ALERT_2_G Hirshfeld Test Diff for S5 -- C14F .. 5.2 su
PLAT301_ALERT_3_G Main Residue Disorder .... Percentage = 6 Note
PLAT605_ALERT_4_G Structure Contains Solvent Accessible VOIDS of . 97 A**3
PLAT720_ALERT_4_G Number of Unusual/Non-Standard Labels .... 16 Note
PLAT779_ALERT_4_G Suspect or Irrelevant (Bond) Angle in CIF .... # 13 Check
S1 -C3 -S1M 1.555 1.555 1.555 32.68 Deg.
PLAT779_ALERT_4_G Suspect or Irrelevant (Bond) Angle in CIF .... # 77 Check
S4 -C3' -S4' 1.555 1.555 1.555 5.20 Deg.
PLAT779_ALERT_4_G Suspect or Irrelevant (Bond) Angle in CIF .... # 267 Check
S5N -C3F -S5 1.555 1.555 1.555 26.97 Deg.
PLAT791_ALERT_4_G The Model has Chirality at C3D .... S Verify
PLAT791_ALERT_4_G The Model has Chirality at C5 .... R Verify
PLAT791_ALERT_4_G The Model has Chirality at C5F .... R Verify
PLAT791_ALERT_4_G The Model has Chirality at C6' .... S Verify
PLAT791_ALERT_4_G The Model has Chirality at C6D .... S Verify
PLAT791_ALERT_4_G The Model has Chirality at C10B .... S Verify
PLAT791_ALERT_4_G The Model has Chirality at C10D .... S Verify
PLAT791_ALERT_4_G The Model has Chirality at C11A .... S Verify
PLAT791_ALERT_4_G The Model has Chirality at C11C .... S Verify
PLAT860_ALERT_3_G Number of Least-Squares Restraints .... 47 Note
PLAT869_ALERT_4_G ALERTS Related to the use of SQUEEZE Suppressed ! Info

```
PLAT899_ALERT_4_G SHELXL97   is Deprecated and Succeeded by SHELXL       2014 Note
```

```
  1 ALERT level A = Most likely a serious problem - resolve or explain
  8 ALERT level B = A potentially serious problem, consider carefully
 13 ALERT level C = Check. Ensure it is not caused by an omission or oversight
 26 ALERT level G = General information/check it is not something unexpected

  2 ALERT type 1 CIF construction/syntax error, inconsistent or missing data
 19 ALERT type 2 Indicator that the structure model may be wrong or deficient
  7 ALERT type 3 Indicator that the structure quality may be low
 18 ALERT type 4 Improvement, methodology, query or suggestion
  2 ALERT type 5 Informative message, check
```

It is advisable to attempt to resolve as many as possible of the alerts in all categories. Often the minor alerts point to easily fixed oversights, errors and omissions in your CIF or refinement strategy, so attention to these fine details can be worthwhile. In order to resolve some of the more serious problems it may be necessary to carry out additional measurements or structure refinements. However, the purpose of your study may justify the reported deviations and the more serious of these should normally be commented upon in the discussion or experimental section of a paper or in the "special_details" fields of the CIF. checkCIF was carefully designed to identify outliers and unusual parameters, but every test has its limitations and alerts that are not important in a particular case may appear. Conversely, the absence of alerts does not guarantee there are no aspects of the results needing attention. It is up to the individual to critically assess their own results and, if necessary, seek expert advice.

Publication of your CIF in IUCr journals

A basic structural check has been run on your CIF. These basic checks will be run on all CIFs submitted for publication in IUCr journals (*Acta Crystallographica*, *Journal of Applied Crystallography*, *Journal of Synchrotron Radiation*); however, if you intend to submit to *Acta Crystallographica Section C* or *E*, you should make sure that full publication checks are run on the final version of your CIF prior to submission.

Publication of your CIF in other journals

Please refer to the *Notes for Authors* of the relevant journal for any special instructions relating to CIF submission.

PLATON version of 20/08/2014; check.def file version of 18/08/2014

Datablock g__140718d_work_cu_140718d_0m - ellipsoid plot

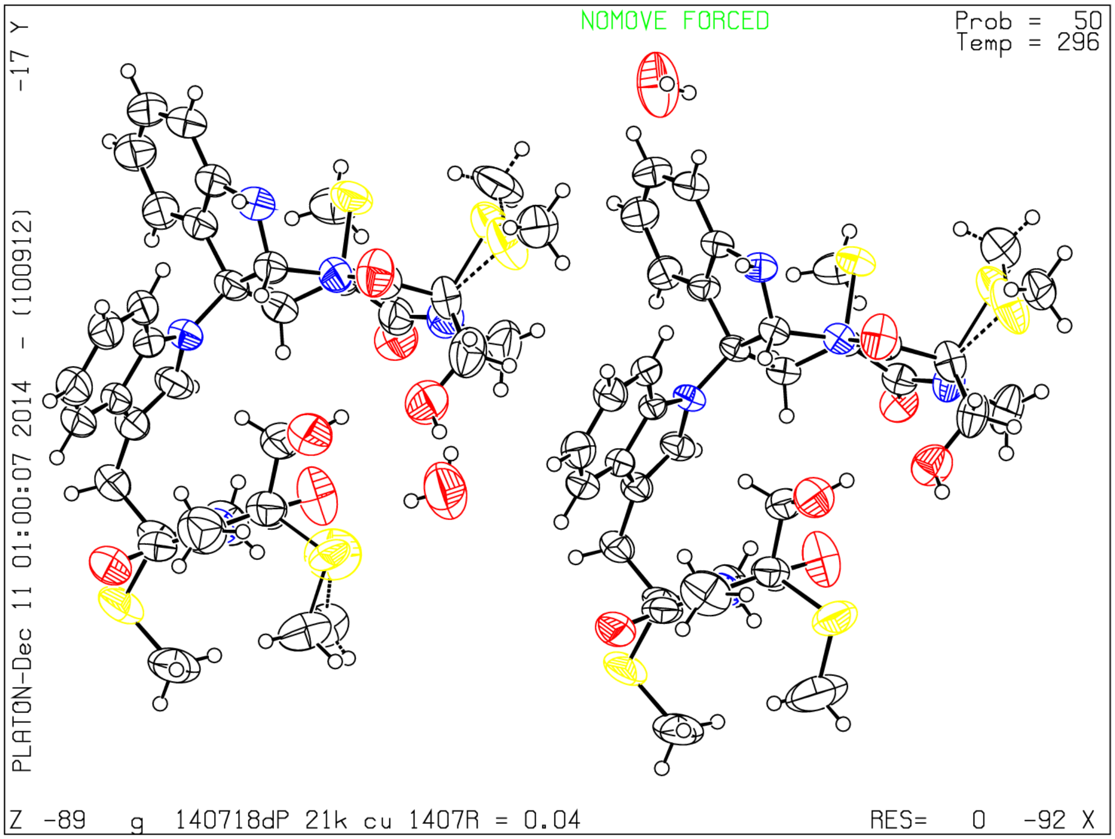

Supplement: Supplementary Information — CIF check of Chaetocochins G (1) [file srep09294-s2.pdf]
