# Supplementary material for: Indole diketopiperazines from endophytic Chaetomium sp 88194 induce breast cancer cell apoptotic death
Source: Sci Rep. 2015 Mar 19;5:9294. doi: 10.1038/srep09294 (PMC4365412; doi:10.1038/srep09294)

checkCIF/PLATON report

Structure factors have been supplied for datablock(s) cu_140701d_0m

THIS REPORT IS FOR GUIDANCE ONLY. IF USED AS PART OF A REVIEW PROCEDURE FOR PUBLICATION, IT SHOULD NOT REPLACE THE EXPERTISE OF AN EXPERIENCED CRYSTALLOGRAPHIC REFEREE.

No syntax errors found. CIF dictionary Interpreting this report

Datablock: cu_140701d_0m

```
Bond precision:  C-C = 0.0028 A          Wavelength=1.54178

Cell:            a=8.8404(2)    b=14.4203(3)      c=13.5625(2)
                 alpha=90       beta=92.824(1)    gamma=90
Temperature:     298 K
```

	Calculated	Reported
Volume	1726.86(6)	1726.86(6)
Space group	P 21	P 21
Hall group	P 2yb	P 2yb
Moiety formula	2(C16 H19 N3 O4 S), H2 O	2(C16 H20 N3 O4 S), H2 O
Sum formula	C32 H40 N6 O9 S2	C32 H40 N6 O9 S2
Mr	716.82	716.82
Dx,g cm-3	1.379	1.379
Z	2	2
Mu (mm-1)	1.925	1.925
F000	756.0	756.0
F000'	759.63	
h,k,lmax	10,17,16	10,17,16
Nref	6395[3335]	6000
Tmin,Tmax	0.338,0.463	0.700,0.700
Tmin'	0.222	

```
Correction method= MULTI-SCAN

Data completeness= 1.80/0.94      Theta(max)= 68.720

R(reflections)= 0.0284( 5934)     wR2(reflections)= 0.0787( 6000)

S = 1.042                   Npar= 452
```

The following ALERTS were generated. Each ALERT has the format
test-name_ALERT_alert-type_alert-level.
Click on the hyperlinks for more details of the test.

Alert level C

```
PLAT029_ALERT_3_C _diffrn_measured_fraction_theta_full Low .......      0.969 Note
PLAT089_ALERT_3_C Poor Data / Parameter Ratio (Zmax < 18) ........       7.38 Note
PLAT220_ALERT_2_C Large Non-Solvent  C     Ueq(max)/Ueq(min) Range        3.4 Ratio
PLAT230_ALERT_2_C Hirshfeld Test Diff for    N27    --  C26     ..        5.7 su
PLAT242_ALERT_2_C Low       Ueq as Compared to Neighbors for .....         S2 Check
PLAT355_ALERT_3_C Long    O-H (X0.82,N0.98A) O1S    -   H1SB   ...       1.04 Ang.
PLAT417_ALERT_2_C Short Inter D-H..H-D       H1SB   ..  H34     ..       2.12 Ang.
PLAT420_ALERT_2_C D-H Without Acceptor       N22    -   H22    ...     Please Check
```

Alert level G

```
FORMU01_ALERT_1_G  There is a discrepancy between the atom counts in the
            _chemical_formula_sum and _chemical_formula_moiety. This is
            usually due to the moiety formula being in the wrong format.
            Atom count from _chemical_formula_sum:   C32 H40 N6 O9 S2
            Atom count from _chemical_formula_moiety:C32 H42 N6 O9 S2
PLAT005_ALERT_5_G No _iucr_refine_instructions_details  in the CIF     Please Do !
PLAT007_ALERT_5_G Number of Unrefined Donor-H Atoms .............         10 Report
PLAT033_ALERT_4_G Flack x Value Deviates > 2*sigma from Zero .....      0.025
PLAT042_ALERT_1_G Calc. and Reported MoietyFormula Strings  Differ     Please Check
PLAT063_ALERT_4_G Crystal Size Likely too Large for Beam Size ....       0.71 mm
PLAT093_ALERT_1_G No su's on H-positions, refinement reported as .      mixed
PLAT380_ALERT_4_G Incorrectly? Oriented X(sp2)-Methyl Moiety .....        C16 Check
PLAT720_ALERT_4_G Number of Unusual/Non-Standard Labels ..........          2 Note
PLAT791_ALERT_4_G The Model has Chirality at C3      .............          S Verify
PLAT791_ALERT_4_G The Model has Chirality at C6      .............          R Verify
PLAT791_ALERT_4_G The Model has Chirality at C20     .............          S Verify
PLAT791_ALERT_4_G The Model has Chirality at C23     .............          R Verify
PLAT899_ALERT_4_G SHELXL97   is Deprecated and Succeeded by SHELXL       2014 Note
```

---

0 **ALERT level A** = Most likely a serious problem - resolve or explain
0 **ALERT level B** = A potentially serious problem, consider carefully
8 **ALERT level C** = Check. Ensure it is not caused by an omission or oversight
14 **ALERT level G** = General information/check it is not something unexpected

3 ALERT type 1 CIF construction/syntax error, inconsistent or missing data
5 ALERT type 2 Indicator that the structure model may be wrong or deficient
3 ALERT type 3 Indicator that the structure quality may be low
9 ALERT type 4 Improvement, methodology, query or suggestion
2 ALERT type 5 Informative message, check

It is advisable to attempt to resolve as many as possible of the alerts in all categories. Often the minor alerts point to easily fixed oversights, errors and omissions in your CIF or refinement strategy, so attention to these fine details can be worthwhile. In order to resolve some of the more serious problems it may be necessary to carry out additional measurements or structure refinements. However, the purpose of your study may justify the reported deviations and the more serious of these should normally be commented upon in the discussion or experimental section of a paper or in the "special_details" fields of the CIF. checkCIF was carefully designed to identify outliers and unusual parameters, but every test has its limitations and alerts that are not important in a particular case may appear. Conversely, the absence of alerts does not guarantee there are no aspects of the results needing attention. It is up to the individual to critically assess their own results and, if necessary, seek expert advice.

Publication of your CIF in IUCr journals

A basic structural check has been run on your CIF. These basic checks will be run on all CIFs submitted for publication in IUCr journals (*Acta Crystallographica*, *Journal of Applied Crystallography*, *Journal of Synchrotron Radiation*); however, if you intend to submit to *Acta Crystallographica Section C* or *E*, you should make sure that full publication checks are run on the final version of your CIF prior to submission.

Publication of your CIF in other journals

Please refer to the *Notes for Authors* of the relevant journal for any special instructions relating to CIF submission.

PLATON version of 20/08/2014; check.def file version of 18/08/2014

Datablock cu_140701d_0m - ellipsoid plot

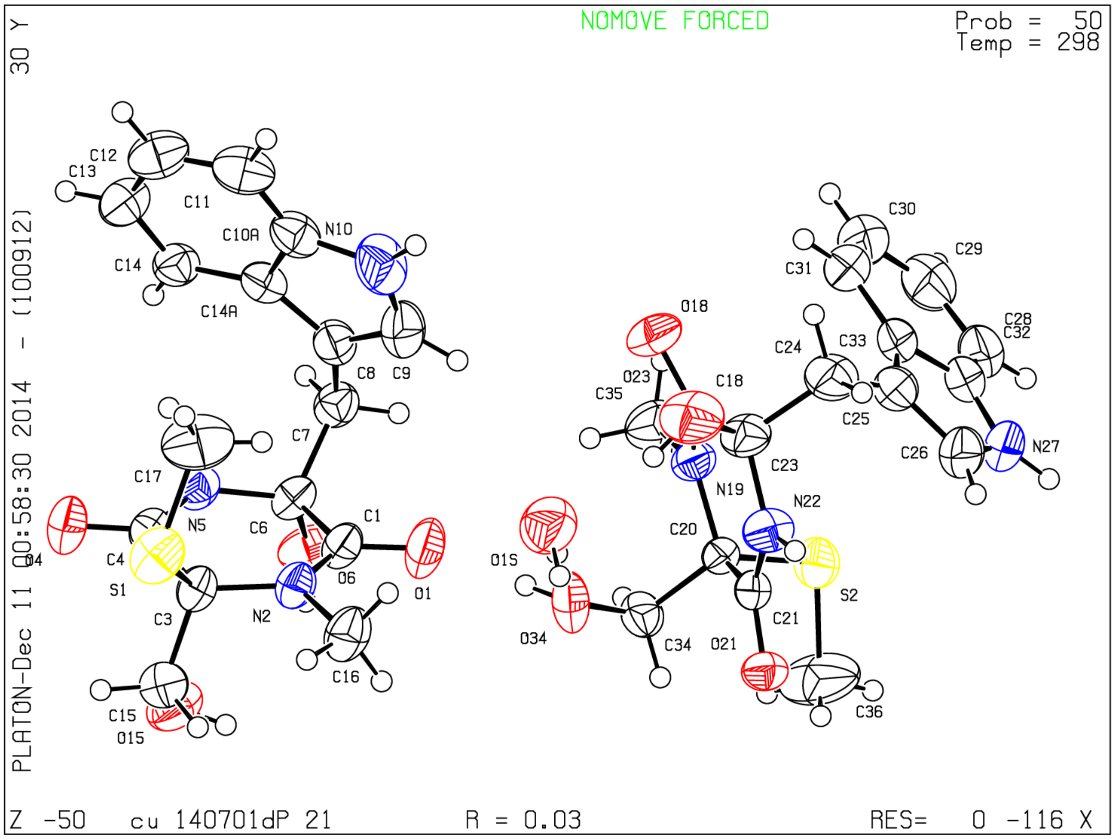

Supplement: Supplementary Information — CIF check of Chetoseminudin E (3) [file srep09294-s3.pdf]
